# Comparative Transcriptomics During Brown Rot Decay in Three Fungi Reveals Strain-Specific Degradative Strategies and Responses to Wood Acetylation

**DOI:** 10.3389/ffunb.2021.701579

**Published:** 2021-09-06

**Authors:** Martina Kölle, Maria Augusta Crivelente Horta, J. Philipp Benz, Annica Pilgård

**Affiliations:** ^1^Chair of Wood Science, TUM School of Life Sciences Weihenstephan, Technical University of Munich, Munich, Germany; ^2^Professorship of Fungal Biotechnology in Wood Science, TUM School of Life Sciences Weihenstephan, Technical University of Munich, Munich, Germany; ^3^Institute of Advanced Study, Technical University of Munich, Munich, Germany; ^4^Biobased Materials, Bioeconomy, RISE Research Institutes of Sweden, Borås, Sweden

**Keywords:** *Rhodonia placenta*, *Postia placenta*, *Gloeophyllum trabeum*, brown rot decay, transcriptome comparison, wood degradation and deterioration, acetylated wood

## Abstract

Brown rot fungi degrade wood in a two-step process in which enzymatic hydrolysis is preceded by an oxidative degradation phase. While a detailed understanding of the molecular processes during brown rot decay is mandatory for being able to better protect wooden products from this type of degradation, the underlying mechanisms are still not fully understood. This is particularly true for wood that has been treated to increase its resistance against rot. In the present study, the two degradation phases were separated to study the impact of wood acetylation on the behavior of three brown rot fungi commonly used in wood durability testing. Transcriptomic data from two strains of *Rhodonia placenta* (FPRL280 and MAD-698) and *Gloeophyllum trabeum* were recorded to elucidate differences between the respective decay strategies. Clear differences were found between the two decay stages in all fungi. Moreover, strategies varied not only between species but also between the two strains of the same species. The responses to wood acetylation showed that decay is generally delayed and that parts of the process are attenuated. By hierarchical clustering, we could localize several transcription factors within gene clusters that were heavily affected by acetylation, especially in *G. trabeum*. The results suggest that regulatory circuits evolve rapidly and are probably the major cause behind the different decay strategies as observed even between the two strains of *R. placenta*. Identifying key genes in these processes can help in decay detection and identification of the fungi by biomarker selection, and also be informative for other fields, such as fiber modification by biocatalysts and the generation of biochemical platform chemicals for biorefinery applications.

## Introduction

Brown rot fungi occur naturally within the northern coniferous forest ecosystems, where they also represent the most dominant form of wood decay (Eriksson et al., 1990; Blanchette, 1991; Daniel, 1994). Due to their preference of growing mainly on softwood, they are one of the most important microorganisms when it comes to degradation of wooden products (Zabel and Morrell, 1992; Rowell, 2005). Brown rot degradation leads to massive strength loss through depolymerization of the cellulose fraction before mass loss is even detectable (Eaton and Hale, 1993; Goodell, 2003). Previous research suggests that brown rot degrades wood in a two-step mechanism (Goodell, 2003; Baldrian and Valášková, 2008; Arantes et al., 2012; Zhang et al., 2016). Oxidative, non-enzymatic processes solubilize sugars leading to the secretion of hydrolytic enzymes (Curling et al., 2002; Filley et al., 2002; Martinez et al., 2005; Niemenmaa et al., 2008; Arantes et al., 2012; Zhang et al., 2016; Zhang and Schilling, 2017). The currently most accepted course of action is as follows: Oxalic acid, secreted by the brown-rot fungi, diffuses through the cell wall into the lumen, where it chelates Fe^3+^ (Goodell et al., 1997; Eastwood et al., 2011; Arantes et al., 2012). Through reduction by hydroquinones and other reducing agents, Fe^2+^ is formed, which reacts with H_2_O_2_, likely generated by a reaction between oxygen and hydroquinone's (Paszczynski et al., 1999; Jensen et al., 2001). This step is key to the “chelator-mediated Fenton” reaction (CMF) producing hydroxyl radicals, which depolymerize cellulose and hemicellulose and are able to modify lignin (Fenton, 1894; Baldrian and Valášková, 2008; Arantes et al., 2012). Solubilized sugars diffuse into the plant cell lumen, where they become accessible for cellulases and hemicellulases (Martinez et al., 2005; Goodell et al., 2017). Since brown rot fungi evolved several times independently from white rot fungi, versatile mechanisms for polysaccharide degradation exist, meaning that different brown rot species use variable enzymatic cocktails during the degradation process. Compared to the molecular processes in white rot fungi, brown rot degradation seems to be more complex, as for example more recent studies found that the oxidative phase, driven by reactive oxygen species is also supported by GHs seemingly tolerating oxidative stress (Castaño et al., 2018). However, the details are still not fully understood, showing that more research is needed.

To protect wooden products from degradation and prolong service life, wood modification is an approach to substitute traditionally used copper-based preservatives (Hill, 2006). Acetylation is one type of modification (Hill, 2006) that has been widely studied (Hill, 2006; Alfredsen et al., 2016; Hosseinpourpia and Mai, 2016; Mantanis, 2017; Beck et al., 2018; Zelinka et al., 2020). It has been shown that acetylation enhances the resistance of wooden products against fungal decay, but the exact mode of action remains vague beyond that. The replacement of hydroxyl groups in the cell wall polysaccharides by acetyl groups is thought to create a bulking effect of the cell walls that reduces water absorption through reduced hygroscopicity and volumetric swelling (Rowell, 2005; Hill, 2006; Thybring, 2013; Popescu et al., 2014; Beck et al., 2017). One likely theory considers that the equilibrium moisture content of the wood is reduced by the acetylation, resulting in the inhibition of diffusion of fungal degradation agents through the wood cell wall (Papadopoulos and Hill, 2002; Hill, 2006; Jakes et al., 2013; Ringman et al., 2014; Xie et al., 2015; Zelinka et al., 2016). The inhibition of diffusion has been further observed by several studies on either ion mobility (Hunt et al., 2018), hydrolytic reductants (Ringman et al., 2014) or an interrupted moisture network (Zelinka et al., 2008, 2015, 2016; Beck et al., 2017).

It has been shown that degradation is delayed in modified wood, but once it has started, it appears to progress in a similar manner (Alfredsen et al., 2016; Beck et al., 2018). Since the accumulated data point to the initial decay phase as the crucial step where degradation is inhibited, a better understanding of how and where the fungus is inhibited is mandatory to further improve modification methods. Similarly important for an increased use of wood in construction is the development of biomarkers for detection and monitoring of fungal decay in untreated and modified wood (Gelhaye and Morel, 2009).

Here, we investigated differential gene expression between three brown rot fungi: two strains of *R. placenta* (Fr.) *Niemelä, K.H. Larss. &* Schigel (also known as *Postia placenta*) FPRL280 and MAD-698, as well as *G. trabeum* (Pers.) *Murrill*, all of them presenting different phenotypes (Thaler et al., 2012; Presley and Schilling, 2017; Kölle et al., 2020). The brown rot fungi *R. placenta* and *G. trabeum* are common inhabitants of forest ecosystems and are also largely responsible for the destructive decay of wooden structures (Niemenmaa et al., 2008). Indeed, they are considered the two major experimental organisms for studies of brown rot decay, representing distantly related species origins of brown rot (Hibbett and Donoghue, 2001; Martinez et al., 2009). According to observations *G. trabeum* and *R. placenta* have differences in the decay mechanisms, as during the demethoxylation reactions (Niemenmaa et al., 2008). In addition, they are largely used in standard wood durability testing (EN113) (CEN, 1996). Our aim was to reveal differences in brown rot degradation strategies to further elucidate the complexity of the ongoing processes during lignocellulose decay. This would be beneficial for fungal biotechnological applications as for example biomass pretreatment in bio refineries as well as the development of novel wood preservation methods. For this, the wafer degradation method (Schilling et al., 2013) was adopted to be able to separate non-enzymatic oxidative degradation from the enzymatic (hydrolytic) degradation phase. A transcriptomics approach was subsequently used to provide insight into differences in gene expression when growing on untreated vs. acetylated wood. Understanding how fungal behavior and growth is affected by wood modifications is important for the optimization and development of new wood protection methods. Furthermore, these findings will also be valuable for the development of new commercial applications for the degradation of lignocellulosic materials. Based on the previous genome comparison of the two *R. placenta* strains (Kölle et al., 2020), differences in overall gene expression should further elaborate the adapted degradation mechanisms between them.

## Materials and Methods

### Wood Material

Wood wafers (80 × 18 × 2.3 mm^3^) of Scots pine sapwood (*Pinus sylvestris*) were cut, dried and weighed (Zhang et al., 2016). Half of the samples were acetylated (15%) according to (Kölle et al., 2019). All samples were autoclaved prior to the decay test.

### Decay Test

Weck jars were filled with a growth medium composed of 50 g soil, 25 g sand, 20 g vermiculite, and 45 mL of water for each sample container. Three feeder strips of pine wood were placed on the medium per glass and inoculated with either *R. placenta* (strain FPRL280), *R. placenta* (strain MAD-698) or *G. trabeum* (strain BAM 115), originally cultivated on potato dextrose agar (*R. placenta*) or 4% malt agar (*G. trabeum*) plates. After the feeder strips were completely overgrown with mycelium, one wafer was placed on each feeder strip. Three glasses per treatment and fungus were prepared. Jars were stored in a climate chamber (25°C, 65% rh) until wafers were ¾ overgrown. Since the growth speed was more or less the same for all strains and treatments, we assumed that these parameters (5 mm section for oxidative decay and ¾ line) are suitable for all three strains. A 5 mm section containing the hyphal front was taken representing the oxidative degradation zone. The rest of the mycelia was used for enzymatic degradation analysis. The section size of 5 mm was chosen due to a growth rate of about 2.5 mm/day, with the first 5 mm thus approximately representing a 48 h window, during which the non-enzymatic degradation phase has been shown to take place (Zhang et al., 2016). All three samples in one glass were pooled into one biological replicate.

### RNA Purification

Samples were ground with an MM400 Mixer Mill (Retsch GmbH, Haan, Germany) using one 1.5 cm steel ball and 30 Hz for 2 min. All equipment was pre-cooled in liquid nitrogen. Sixty mg of wood powder was taken for RNA purification. Total RNA was extracted and purified using the MasterPure^TM^ RNA Purification Kit (Lucigen) (Ringman et al., 2016).

### Sequencing

The quality of RNA extraction was measured with the Agilent 2,100 Bioanalyzer. The TruSeq stranded mRNA Library Prep Kit (Illumina) with TruSeq RNA Single Indexes Set A and Set B was used to generate the RNA-Seq library preparation 500 ng of input-RNA was quantified using a Qubit Fluorometer 2.0 and a Qubit RNA BR Assay Kit (both Invitrogen). Libraries were quantified with a Qubit Fluorometer 2.0 and a Qubit DNA HS Assay Kit. The fragment length of the libraries was reviewed and finally quantified with qPCR [QuantStudio 5 (Applied Biosystems)] according to the Illumina qPCR Guide using KAPA SYBR Fast Mastermix Low Rox (KAPA Biosystems). The libraries were normalized to 2nM with EB (Qiagen) and equally pooled (according to Illumina User Guide). An overview of the fragment lengths for all strains, treatments and decay stages is given in Supplementary Figure 1. The libraries were multiplexed using HiSeq Rapid single-read Cluster Kit v2 and sequenced on the Illumina HiSeq2500 sequencer using a HiSeq Rapid SBS Kit v2 (single-indexed read 1:100 cycles index read for 7 cycles). HiSeq Control Software 2.2.70 was used, image analysis and base calling were performed with Real Time Analysis 1.18.66.4. Fastq-files were generated with the CASAVA BCL2FASTQ Conversion software v2.20.

Transcriptomic data are available on NCBI with the BioProject ID: PRJNA681134 (https://www.ncbi.nlm.nih.gov/sra/PRJNA681134).

### Data Analysis

For data analysis, data were uploaded to the Galaxy Server (Afgan et al., 2018). The reads from all samples were mapped against the total genome using Hisat2 (Kim et al., 2015). For transcript prediction, the mapped reads of each sample were assembled by StringTie (v1.3.3b) (Pertea et al., 2015, 2016) using a reference-based approach. BCFtools was used to perform the variant calling against genome sequences. The variant annotation and the prediction of functional effects were performed with SnpEff v5.0. Gene expression was analyzed using CLC Genomic Workbench v. 20.0.4 (Qiagen). Reads were mapped against the corresponding genomes (Floudas et al., 2012; Kölle et al., 2020), while the MAD-698 transcriptome data were mapped to the monokaryotic genome of MAD-SB12 (Gaskell et al., 2017). Mapping statistics can be found in Supplementary Material 3. MAD-SB12 genome was used as reference following previous results by Kölle et al. (2020). the genome size determined for FPRL280 is similar to the monokaryotic condition of MAD-SB12, isolated from a basidiospore of the fruiting dikaryon MAD-698, (Gaskell et al., 2017). Expression analysis was normalized and measured in TPM Ortholog search obtained the corresponding orthologous genes of all three strains. In total, 9,581 orthologs were found between the two strains of *R. placenta*, 6,145 orthologs between FPRL280 and *G. trabeum* and 6,381 between MAD-SB12 and *G. trabeum* (Supplementary Material 1). The gene set of the two strains with the lowest number of orthologs was chosen to create a list with orthologs to compare all three strains (Supplementary Material 2). Differential expression analysis was performed between the four groups within each strain [untreated/oxidative (UT/Ox); untreated/enzymatic (UT/Enz); acetylated/oxidative (AC/Ox); acetylated/enzymatic (AC/Enz)], using a log2 fold change cutoff of ±2 and a *p*-value of ≤0.05 (Supplementary Materials 2, 3). The differential expression analysis compares the oxidative and the enzymatic state of untreated samples of the two *R. placenta* strains FPRL280 and MAD-698 (Supplementary Material 4). A functional enrichment analysis was done using the Gene Ontology (GO) terms of both *R. placenta* strains and *G. trabeum*. Functional groups were described according to the model of (Zhang et al., 2016). A separate analysis classified brown rot-specific BRS genes into two groups, genes that are active during brown rot decay or have oxidative functions (BRS “Ox”) and those that are related to enzymatic brown rot decay or have functions related to sugar metabolism (as for example GHs) (BRS “Enz”) (Supplementary Material 5). The BRS gene list was assembled based on previous reports from Martinez et al. (2009), Ryu et al. (2011), and Zhang et al. (2016) and was manually accurate with genes with specific functional annotation that showed phase-specific expression behavior. Genes encoding carbohydrate active enzymes (CAZy) were also grouped separately as the following groups: transporters, cytochrome P450 genes (partly putative), proteases and aldo-keto reductases (Supplementary Material 3). Genes with other function were grouped (“others”), including genes encoding uncharacterized or unknown proteins. A Pearson-correlation was performed to correlate MAD-698 data with the published data from (Zhang et al., 2016). Expression levels were hierarchically clustered with the “Hierarchical Clustering Explorer” software v3.5 (Seo and Shneiderman, 2002). Genes with low expression (<10 FPKMs under all conditions) were excluded from the analysis. Data were initially normalized (i.e., the deviation from the mean was divided by the standard deviation). The average linkage method was used for cluster generation, with centered Pearson's correlation as distance/similarity measure. Nodes were arranged with the smaller subtrees kept to the right.

## Results

### Reads Mapping to the Reference Genome

We checked the accuracy of the previously published *R. placenta* FPRL280 genome (Kölle et al., 2020) for the analysis of transcriptomes (Supplementary Material 1). A variance rate of only one variant every 6,926 bases, with a total of 4,576 variant events, was detected for the genome assembly when all transcripts were mapped against the reference *R. placenta* FPRL280, confirming the reliability of the reference genome.

### Overall Expression Analysis

To compare the fungal responses during both degradation stages to the treatments, a differential expression analysis was performed. The total number of genes expressed by each strain for each condition is shown in Supplementary Figure 2. Since the wafer method was adopted from a related study testing the two-step response of *R. placenta* MAD-698 to untreated spruce wood samples (Zhang et al., 2016), we compared the gene expression data and found an acceptable degree of correlation [*r* = 0.56; *p* = 0.00; log2 FC between 0 and 5 mm (Ox) and 15–20 mm (Enz)], considering the differences in the used substrates (Spruce vs. Scots Pine).

**Figure 1:**
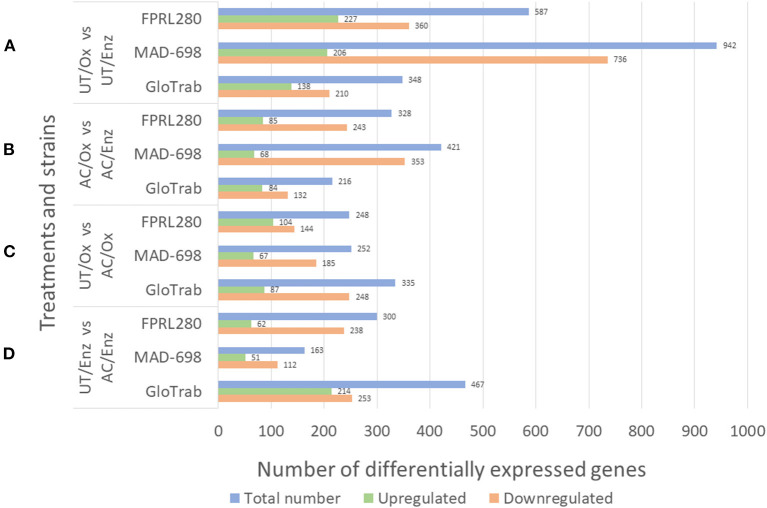
Results for the differential expression analysis of the treatment and decay stage comparison of the three brown rot strains *R. placenta* FPRL280 and MAD-698 and *G. trabeum* (GloTrab). The treatments that were compared are untreated (UT) and 15 % acetylated (AC) samples, at different decay stages: early oxidative decay (Ox) and later enzymatic decay (Enz). **(A)** Shows the comparison of the oxidative (Ox) and the enzymatic (Enz) phase in untreated (UT) samples. **(B)** Compares the oxidative and the enzymatic phase, but in acetylated samples. **(C)** Compares the oxidative phases of untreated and acetylated samples. **(D)** Shows the comparison of untreated and acetylated samples during the enzymatic decay phase.

The results for the differential expression analysis are shown in Figure 1. The differential expression analysis (DE) between UT/Ox and UT/Enz revealed the majority of genes to be upregulated during enzymatic decay (Figure 1A). There were also more genes upregulated in AC/Enz compared to AC/Ox. When comparing untreated and acetylated samples, it can be seen that there are more genes upregulated in acetylated samples, both during oxidative [UT/Ox vs. AC/Ox: FPRL280: 104 vs. 144; MAD-698: 67 vs. 185; GloTrab: 87 vs. 248) and enzymatic decay (UT/Enz vs. AC/Enz: FPRL280: 62 vs. 238; MAD-698: 51 vs. 112; GloTrab: 214 vs. 253]. In total, a higher number of genes were differentially expressed in untreated samples compared to acetylated samples.

### Comparison of the Non-enzymatic Oxidative and the Enzymatic Decay Phases

**Figure 2:**
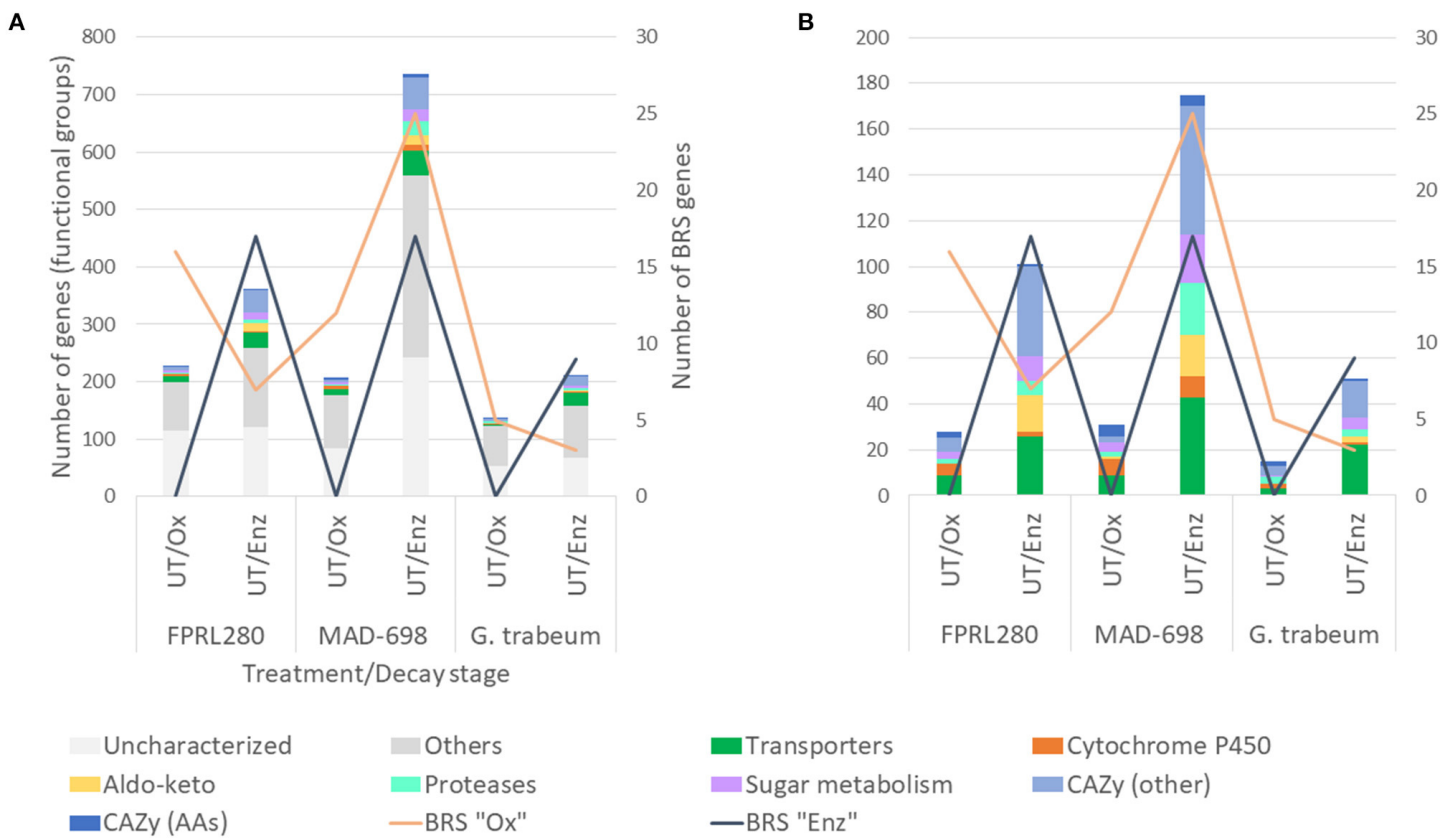
Functional groups of genes significantly upregulated in untreated samples during oxidative and enzymatic brown rot decay. The figure shows the two *R. placenta* strains used for this study (FPRL280 and MAD-698) and *G. trabeum*. **(A)** For the grouping the categories “Transporters”, “Cytochrome P450”, “Aldo-keto” and “Proteases”, genes encoding for enzymes involved in sugar metabolism as well as “CAZy”, “BRS Ox” and “BRS Enz” (brown rot specific) were used. Genes with other functions, as well as uncharacterized genes are also shown. **(B)** For enhanced readability the categories of “Others” and “Uncharacterized” were exluded.

When comparing gene expression profiles between oxidative (early) and enzymatic (late) phase on untreated wood samples, different numbers of expressed genes were revealed between the strains, but with a similar grouping of the associated GO terms (Figure 2).

In all three strains, the number of CAZy-encoding genes and genes encoding proteins involved in sugar metabolism were substantially higher in the enzymatic phase. The total number of differentially expressed brown rot-specific (BRS) genes (Supplementary Material 5) varied between the strains: FPRL280 and *G. trabeum* expressed a higher number of BRS “Ox” genes during the oxidative state, and more BRS “Enz” genes during enzymatic decay. During the oxidative phase, no BRS “Enz” genes were expressed in all three strains in untreated samples. This clearly shows the separation and the delay of the enzymatic phase, as previously suggested by Zhang et al. (2016).

However, in MAD-698 the number of upregulated BRS “Ox” genes was increased during enzymatic decay compared to the oxidative phase. While the number of upregulated BRS “Enz” genes were almost the same in FPRL280 compared to MAD-698 during enzymatic decay, *G. trabeum* expressed the lowest number of genes in both stages. However, this observation can at least partly be attributed to the fact that a lower number of orthologs of *G. trabeum* could be used to find the corresponding BRS genes in this species.

These results show that both strains of *R. placenta* rely on a higher number of genes during enzymatic decay when compared to oxidative decay, as well as when compared to *G. trabeum*. MAD-698 seems to upregulate a more diverse cocktail of genes than FPRL280 during enzymatic decay as well as a higher number of BRS “Ox” genes. All three strains express more BRS “Enz” genes during enzymatic decay.

### CAZy Genes and the Importance of GH5_5 (Cel5A, Cel5B) and GH12 (Cel12A)

**Figure 3:**
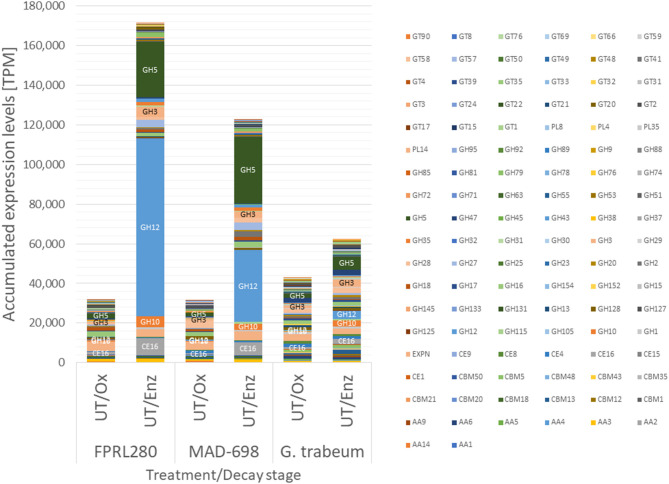
Accumulated expression values [transcripts per million (TPM)] for all CAZy families in strains of *R. placenta* and *G. trabeum* showing the differences between the strains and the decay stages. The largest bar of FPRL280/UT/Enz and MAD-698/UT/Enz is the GH12 family. The second larger group represents the CAZy family GH5. More detailed graphics on the single CAZy families are shown in Supplementary Figures 3A–F.

Since genes encoding CAZymes displayed the most distinct regulatory shift from “Ox” to “Enz” stages (Figure 2), we analyzed their absolute expression levels in more detail (Figure 3, Supplementary Material 3, Supplementary Figures 3A–F).

During oxidative degradation, the expression profiles of CAZy genes of the three strains appeared similar. *G. trabeum* displayed slightly higher accumulated expression levels, which however can be partly attributed to the higher overall number of CAZy genes (352) compared to the *Rhodonia* strains (317). CAZy gene induction during enzymatic decay is then much more pronounced in the *R. placenta* strains. The most highly induced CAZy families were GH12 (predicted endoglucanase and xyloglucan hydrolase functions), GH5 (predicted endo-β-1,4-glucanase/cellulase, endo-β-1,4-xylanase, and β-glucosidases, among other functions), carbohydrate esterase (CE)16 (acetylesterase function on various carbohydrate acetyl esters), GH10 (endo-1,4-β-xylanase and endo-1,3-β-xylanase function) and GH3 (β-glucosidase, xylan 1,4-β-xylosidase, and β-glucosylceramidase predicted functions). In *R. placenta*, particularly one GH12 family member, the endoglucanase Cel12A (FPRL280_170_10; POSPLADRAFT_1071715), contributes largely to this effect with a log2 FC of 7.98 (FPRL280) and 5.92 (MAD-698) between enzymatic and oxidative degradation. Within the GH5 family, several genes are most strongly induced from oxidative to enzymatic decay: GH5_22 (FPRL280_75_15; POSPLADRAFT_1169431), GH5_5 [FPRL280_14_15; POSPLADRAFT_1164613 (Cel5A) and FPRL280_14_16; POSPLADRAFT_1068430 (Cel5B)] and additionally in MAD-698 GH5_7 (POSPLADRAFT_1030481 and POSPLADRAFT_1177854). In *G. trabeum*, one CE16-encoding gene (GLOTRDRAFT_48624) as well as one GH12- (GLOTRDRAFT_138821) and two GH5-encoding genes (GH5_5: GLOTRDRAFT_57704; GH5_7: GLOTRDRAFT_110405) are responsible for the differences, but not to the same level. These results show that all three strains enhance CAZy gene expression during later enzymatic decay. This shift is strongly pronounced in MAD-698 and FPRL280.

### Differences in Decay Strategies Between FPRL280 and MAD-698

**Figure 4:**
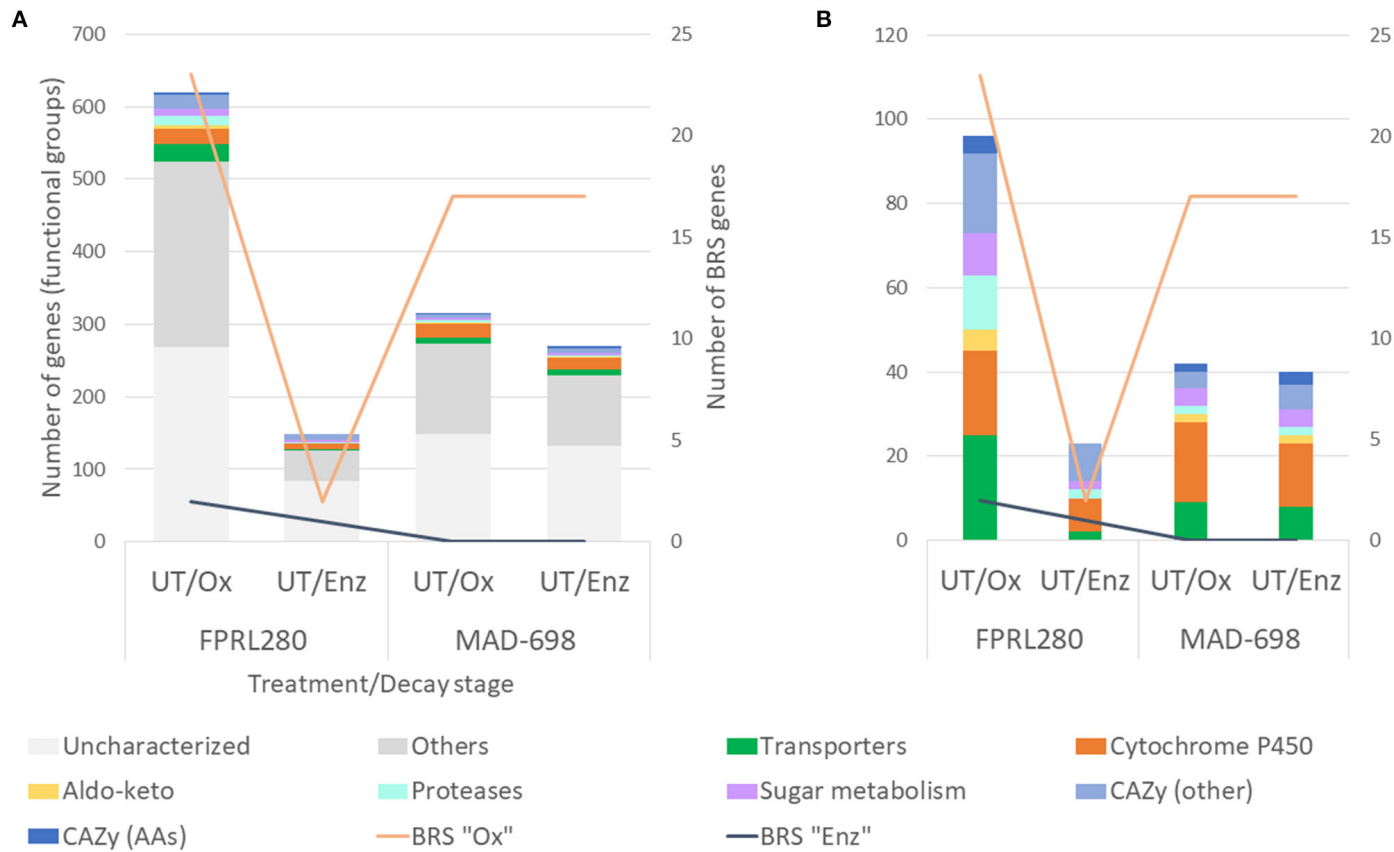
Differential expression analysis, comparing the two *R. placenta* strains during two decay stages on untreated samples: **(A)** Genes, grouped by the gene ontology functional groups: Transporters, Cytochrome P450, CAZys and genes encoding proteins that are involved in sugar metabolism, Aldo-keto reductases, Proteases, genes with other functions and uncharacterized genes. **(B)** Same as in **(A)**, but excluding the groups “Others” and “Uncharacterized”.

Rather minor genomic differences have been observed between the two strains of *R. placenta* (FPRL280 and MAD-698) (Kölle et al., 2020), raising the question whether the phenotypic differences between the strains (Thaler et al., 2012; Kölle et al., 2020) are dominated by transcriptional differences. Therefore, a differential expression analysis of the orthologous genes shared by the *R. placenta* strains was performed (Figure 4). This analysis resulted in 620 genes being upregulated (by over two-fold) by FPRL280 during the oxidative degradation phase compared to MAD-698, while MAD-698 had 315 genes upregulated during oxidative phase. The enzymatic phase showed a reverse trend, since MAD-698 had 270 genes upregulated while only 149 genes were upregulated in FPRL280 during the enzymatic phase.

To determine the genes that differ most in terms of expression levels between the two strains, a log2 fold change cutoff of ±6 was applied (genes that were differentially expressed between the two *R. placenta* strains during oxidative decay can be found in Supplementary Material 5). The cutoff resulted in 26 genes being more highly expressed in FPRL280 vs. MAD-698 during growth on UT/Ox, with two of these genes exceeding thousand twenty four-fold expression. While most of these highly differentially expressed genes are uncharacterized, two proteases and one transporter were among this group. MAD-698 showed 41 genes extensively upregulated vs. FPRL280, one gene encoding a protease, two putative cytochrome P450s and one gene encoding a member of the CAZy family GH13, involved in alpha-amylase activity, calcium ion binding and carbohydrate catabolic processes. In the enzymatic phase of FPRL280, we found 10 genes with a log2 fold change ≥ 6 over MAD-698, four of them fc>10, including one protease-encoding gene and two encoding nucleic acid binding proteins.

In MAD-698, 24 genes were expressed with a fc ≥ 6 in the enzymatic phase, including a cytochrome P450-encoding gene and a GH13 family member. Overall, FPRL280 upregulated a higher number of BRS “Ox” genes during the oxidative phase compared to MAD-698, whereas in MAD-698 these genes are more highly expressed in number during enzymatic decay. FPRL280 upregulated more BRS “Enz” genes during the oxidative and the enzymatic phase compared to MAD-698 (Supplementary Material 5). It is noteworthy that in contrast to FPRL280, MAD-698 upregulated the same number of BRS “Ox” genes during oxidative and enzymatic decay.

In conclusion, FPRL280 and MAD-698 upregulated different genes during enzymatic decay. These differences are observed in CAZy-genes and genes with unknown functions. Furthermore, it could be observed that MAD-698 upregulated a relatively high number of BRS “Ox” genes during enzymatic decay compared to FPRL280.

### The Effect of Wood Acetylation on the Fungal Response

To elucidate how brown rot fungi react to wood acetylation on a genome-wide scale, we next analyzed the differences in gene expression of the same decay phases between treated and untreated wood samples (Supplementary Material 4). FPRL280 downregulated a larger number of genes encoding cytochrome P450s (11), as well as proteases (7) and aldo-keto reductases (3) in acetylated wood samples compared to untreated samples during oxidative decay. No aldo-keto reductases or cytochrome P450 and only one protease was upregulated in acetylated samples compared to untreated samples. In MAD-698, acetylation induced cytochrome P450-encoding genes (6) and, other than in FPRL280, a higher number of transporters (11). *G. trabeum* showed the largest differences between the treatments in the group of genes with “other” functions. Furthermore, *R. placenta* upregulated a higher number of genes encoding for proteins involved in sugar metabolism in acetylated samples during oxidative degradation compared to untreated samples (FPRL280: 12; MAD-698: 5). This was different in *G. trabeum*, where the numbers were equal between the two treatments.

**Figure 5:**
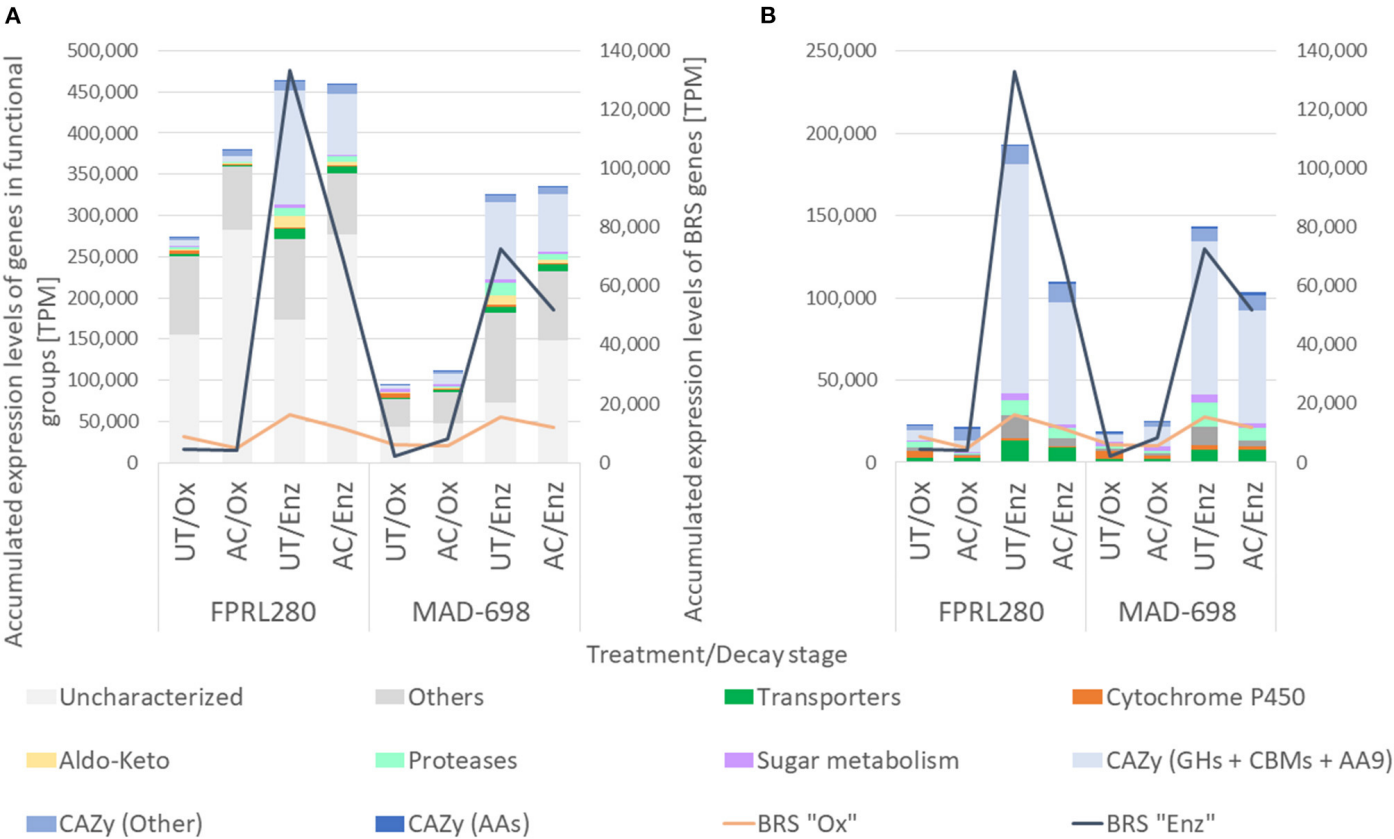
TPMs of the different groups are shown with bars in different colors. **(A)** The graphic shows the results for the differences between untreated and acetylated samples during the oxidative and the enzymatic phase for the two strains of *R. placenta*. *G. trabeum* was excluded due to a better readability of the graphic and can be found in Supplementary Figure 4A. **(B**) To enhance the readability, the groups of “Uncharacterized” and “Others” were excluded. A more detailed view on the single gene groups can be found in Supplementary Figures 4B–M.

The TPMs of all genes of the differential expression analysis were accumulated to see differences in expression levels between the treatments in each functional group (Figure 5; for *G. trabeum* and a group-wise separation see Supplementary Figures 4A–M). Overall, FPRL280 expression levels were higher in acetylated samples during oxidative decay, but similar in the enzymatic phase, compared to untreated samples. While members of the CAZy families GH and AA9, as well as carbohydrate binding modules (CBMs) were not strongly affected during oxidative decay, one expansin-encoding gene (FPRL280_145_15) was clearly upregulated on acetylated wood. In the later enzymatic decay stage, FPRL280 exhibited a lower expression of genes encoding transporters, proteases, aldo-keto reductases, cytochrome P450s and proteins involved in sugar metabolism on acetylated samples compared to untreated samples. The expression levels of CAZy-encoding genes (GH + CBM + AA9) were reduced to about half in acetylated samples compared to untreated samples during enzymatic decay of FPRL280, similar to the levels of BRS “Enz” genes.

In MAD-698, the accumulated TPMs of differentially expressed genes were generally lower than in FPRL280 during both phases, mainly caused by genes of unknown function. As in FPRL280, expression levels of cytochrome P450-encoding genes were lower in acetylated samples during oxidative decay compared to untreated samples, but proteases and transporters were expressed more strongly. POSPLADRAFT_1071715 (Cel12A/GH5_5) is the protein that mainly causes this difference. It is noteworthy that the overall expression level of BRS “Enz” genes during enzymatic degradation was substantially lower in FPRL280 and MAD-698, when comparing acetylated to untreated samples.

Gene expression in *G. trabeum* was overall significantly lower than both strains of *R. placenta* during enzymatic decay, but during oxidative decay, expression levels were intermediate. Lower levels of gene expression were observed for cytochrome P450-, protease- and aldo-keto reductase-encoding genes as well as genes encoding for proteins involved in sugar metabolism during oxidative decay on acetylated samples, compared to untreated samples. The group of “CAZy (Other)”-encoding genes behaved differently when compared to the other two strains, showing lower levels of gene expression in acetylated samples during oxidative decay when compared to untreated samples. Also, genes encoding for AAs did not behave equally in all three strains. *G. trabeum* and FPRL280 showed higher expression levels in AC/Ox samples compared to UT/Ox, while MAD-698 showed the opposite. All three strains have in common that uncharacterized genes represent a large part of the accumulated TPMs, especially in acetylated samples. The genes that most differed between untreated and acetylated samples during oxidative decay in all three strains are listed in Supplementary Material 4.

In total, all three strains expressed higher levels of gene transcripts during initial brown rot degradation in acetylated samples, but similar levels during enzymatic degradation in both treatments. In FPRL280 and *G. trabeum* the differences between UT/Ox and AC/Ox were mainly caused by uncharacterized genes and were larger than in MAD-698. In contrast, the differences between the treatments during enzymatic decay were driven to a larger extent by the functional groups of GHs, CBMs and AA9, especially in *R. placenta*. In all three strains, the expression of BRS “Ox” genes was lower in acetylated samples, reaching out highest levels in UT/Enz samples. The expression of BRS “Enz” genes differed during oxidative decay, but was significantly higher in untreated samples during enzymatic decay in all three strains compared to acetylated samples.

### Hierarchical Clustering and Transcription Factors

**Figure 6:**
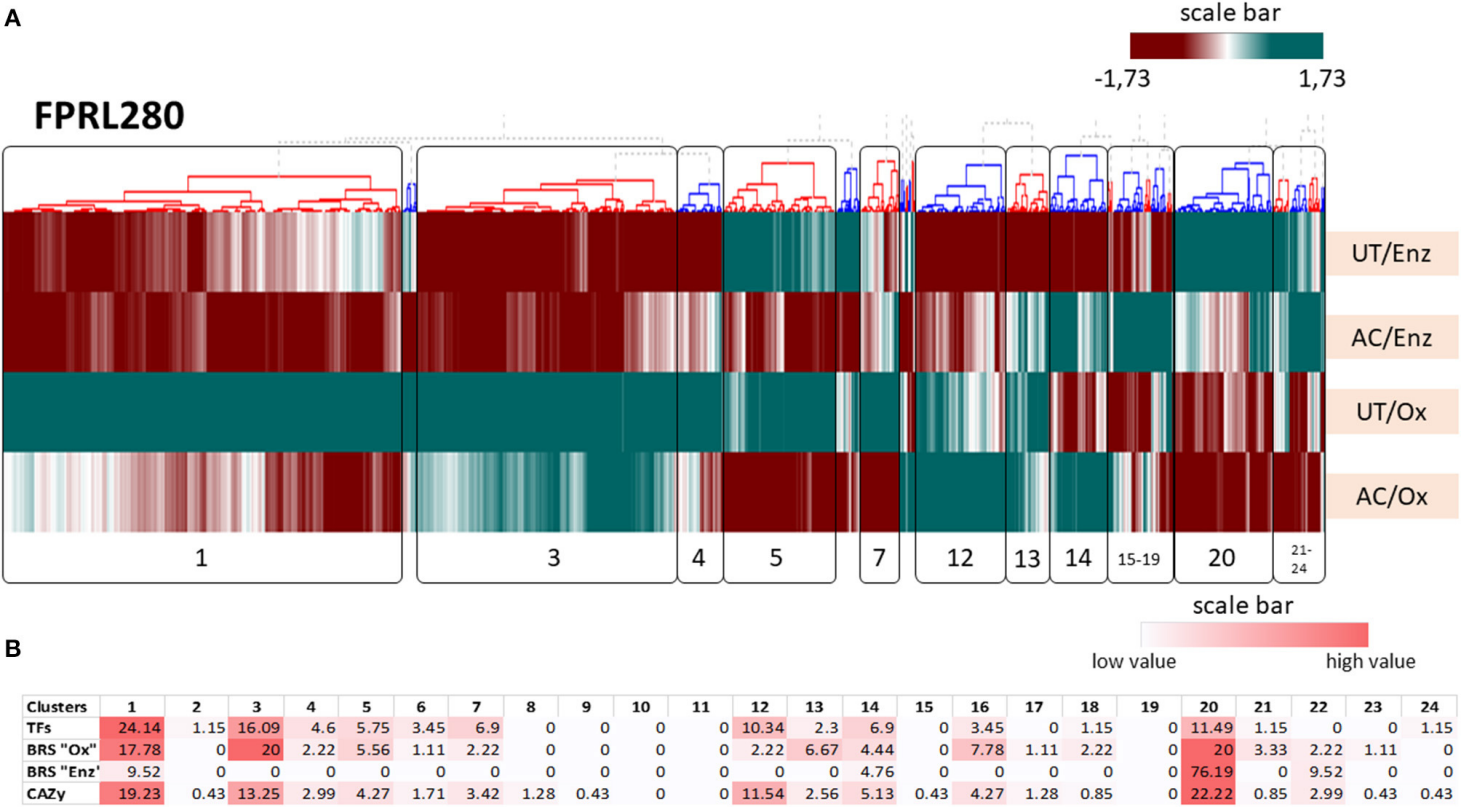
Results for the hierarchical clustering of the transcriptomes of *R. placenta* FPRL280 in all tested conditions. **(A)** UT/Enz: untreated samples during enzymatic decay; AC/Enz: acetylated samples during enzymatic decay; UT/Ox: untreated samples during oxidativ e decay; AC/Ox: acetylated samples during oxidative decay. **(B)** Heat map of brown rot specific genes (BRS), CAZy genes and transcription factors (TFs). The numbers represent the percentage [%] of the abundance of genes of the respective group. To annotate TFs, different references (Martinez et al., 2009; Zhang et al., 2019, UniProt, MycoCosm) were used and the respective orthologs from *R. placenta* and *G. trabeum* identified.

To achieve an additional overview of gene expression profiles between conditions, hierarchical clustering of the whole transcriptomes of *R. placenta* (FPRL280 and MAD-698) and *G. trabeum* was performed, resulting in 24 clusters for FPRL280, 23 clusters for MAD-698 and 16 clusters for *G. trabeum* (Figure 6, Supplementary Material 7 and Supplementary Figures 5A,B).

In FPRL280 clustering, four interesting clusters were identified clustering together genes involved in oxidative decay partly or heavily affected by acetylation (cluster 1), oxidative decay-specific genes that were only slightly affected by acetylation (cluster 3), genes specific for oxidative decay that were enhanced by acetylation (cluster 12) and genes of the enzymatic phase that were affected by the acetylation (cluster 20). These clusters also contained the majority of BRS- and CAZy-encoding genes as well as transcription factors. Cluster 1 contained a high number of genes encoding for GH and GT proteins, as well as the AA3_2 family. Cluster 3 contained several transcription factors (TFs) and BRS “Ox” genes, some of them likely involved in oxalate production. Many GT-encoding genes were located in this cluster. Cluster 20 contained 20% of BRS “Ox”- and the majority of BRS “Enz”-genes, including two genes encoding the endoglucanases Cel12A (GH12) and Cel5A (GH5_5). The clustering results for MAD-698 and *G. trabeum* can be found in Supplementary Material 7. Clustering showed four large gene clusters of FRPL280 that were affected by acetylation, resulting in decreased levels of gene expression in acetylated samples (clusters 1, 3, 20) or in enhanced levels (cluster 12). The clusters 1 and 3 contained genes encoding for proteins of the CAZy families GH, GT and AA3_2 as well as BRS genes. Cluster 3 contained genes that are likely involved in oxalate production, indicating that the acetylation affects oxalate synthesis and thereby possibly indirectly the Fenton reaction. The cluster with highest numbers of BRS genes was cluster 20. Genes located in this cluster encode members of the GH family, including the two endoglucanases Cel5A and Cel12A. In MAD-698 the two endoglucanases-encoding genes (POSPLADRAFT_1164613; POSPLADRAFT_1071715) clustered together with one important gene for oxalate production, an oxalate decarboxylase (OxaD, POSPLADRAFT_ 1058890), strongly affected by the acetylation.

Three specific TFs (FPRL280: 384_5, 28_39 and 10_10), previously shown to be associated with the early oxidative degradation phase in MAD-698 (Zhang et al., 2019), behaved differently in all three fungi on both untreated and acetylated wood. Another Zn(II)2Cys6-type TF associated with early oxidative degradation (Zhang et al., 2019) (FPRL280_11_82, POSPLADRAFT_1043794, GLOTRDRAFT_135433), was detected specifically in the oxidative stage in untreated samples and all strains, but was expressed in acetylated samples only for MAD-698. It is noteworthy that these TFs clustered closely to other proteins potentially related to the degradative process in all three strains, as for example closely to genes encoding an expansin (EXPN), GH128 family members (FPRL280_11_82; POSPLADRAFT_1043794), as well as genes encoding for members of the GH71, GH18, PL14_4, and GH5_9 (GLOTRDRAFT_135433). Another TF (FPRL280_28_39, POSPLADRAFT_1174375) clustered closely to a BRS “Ox” gene encoding an oxaloacetate acetylhydrolase (OahA), a GT69 and a CBM48 and in MAD-698 to a GT48, CBM50 and an EXPN. In *G. trabeum* (GLOTRDRAFT_122681) it clustered together with the BRS “Ox” gene. The TF FPRL280_10_10 clustered closely together with a histidine kinase-encoding gene, a BRS “Ox” gene, a GH16 gene and a second TF, while in MAD-698 this TF (POSPLADRAFT_ 1043897) clustered with a CBM48 and an AA3_2, and in *G. trabeum* (GLOTRDRAFT_ 137651) it clustered with gene coding for GH5_12, GT2, BRS “Ox” and a CBM50. Details on the gene IDs can be found in Supplementary Material 7.

### Important Genes During Brown Rot Decay

**Figure 7:**
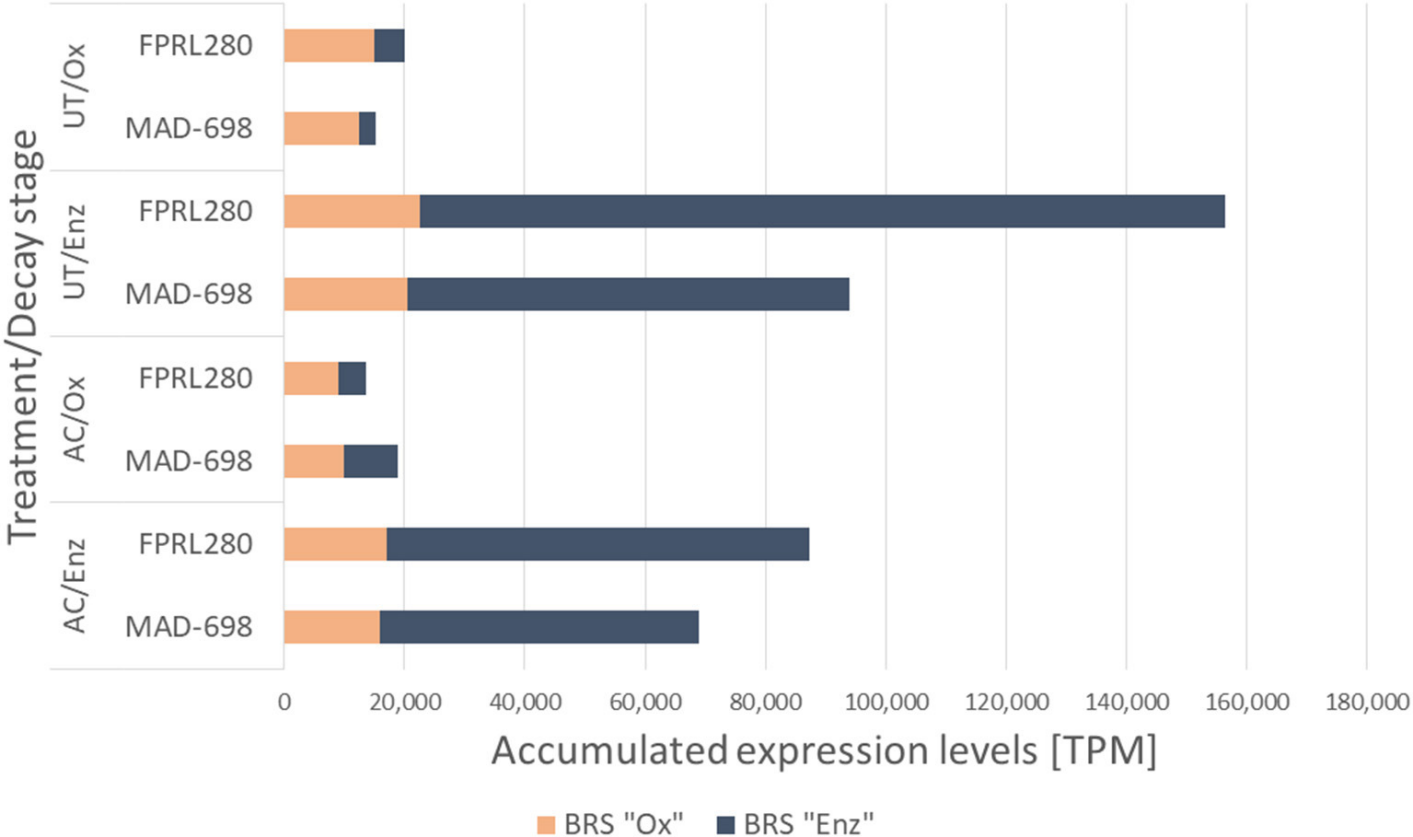
Accumulated expression values for the brown rot specific genes of the two *R. placenta* strains FPRL280 and MAD-698 for all four treatments.

To identify the principal molecular differences during brown rot decay between the two *R. placenta* strains, we next analyzed the accumulated TPMs of brown rot-specific (BRS) genes between the tested conditions (Figure 7). In previous studies on brown rot, several genes had been proposed to be involved in the degradation mechanisms by either oxidative (BRS “Ox”) or enzymatic (BRS “Enz”) action. We collected these genes and created a new list including genes with interesting expression patterns in our analyses (Supplementary Material 5). As was previously observed (Figure 2), significantly higher expression of BRS genes was detected during the enzymatic phase in both strains as well as in both treatments, mainly driven by the high transcription levels of enzymatic BRS genes. From this data, it could also be seen that even though more differentially expressed BRS “Ox” genes are present in MAD-698 (Figure 2: FPRL280/UT/Enz vs. MAD-698/UT/Enz), the accumulated expression levels were actually higher in FPRL280 (Figure 7).

In the majority of cases, BRS genes were expressed at a lower level in acetylated samples compared to untreated samples by all strains, except for BRS “Enz” genes in MAD-698 during oxidative decay, which displayed a slightly elevated expression.

To identify genes that are consistently upregulated during either the oxidative or enzymatic decay stages, the combined results of the differential expression analysis for all three fungi were used. This resulted in a list of six genes being significantly upregulated in oxidative decay samples and 19 being upregulated during enzymatic decay in all three strains (Table 1). To further enlarge the list of genes, the orthologs were used to identify protein names of genes that were already characterized in one of the three strains (UniProt).

**Table 1:** Genes that are significantly upregulated during either oxidative or enzymatic decay in all three strains *R. placenta* FPRL280, MAD-698 and *G. trabeum* with their protein names.

**FPRL280-Id** **FPRL280_**	**MAD-698-Id (SB12) POSPLADRAFT_**	**GloTrab-Id** **GLOTRDRAFT_**	**Protein name**
**UPREGULATED IN ALL THREE STRAINS DURING OXIDATIVE DECAY**			
120_7	1034711	107870	Uncharacterized protein
1_189	1165052	53242	Aspartate aminotransferase
39_6	1146207	80687	Serine/threonine-protein kinase
67_24	1151609	62779	Cyclin N-terminal domain-containing protein
70_40	1058125	111949	Uncharacterized protein
8_99	1037719	58342	Uncharacterized protein
**UPREGULATED IN ALL THREE STRAINS DURING ENZYMATIC DECAY**			
122_7	1067481	116093	MFS domain-containing protein
129_4	1064649	110464	Uncharacterized protein
14_15	1164613	57704	Glycoside hydrolase family 5, Cel5A
170_10	1071715	138821	Glycoside hydrolase family 12, Cel12A
21_14	1072224	76822	Aldo_ket_red domain-containing protein
220_7	1043996	46499	Glycoside hydrolase family 10, Beta-xylanase
221_13	1127293	108929	Uncharacterized protein
28_46	1156080	116882	Uncharacterized protein
327_2	1065808	46545	Carbohydrate esterase family 15
47_21	1047078	105888	Uncharacterized protein
49_4	1174812	69843	Glycoside hydrolase family 3
4_37	1044461	96567	COesterase domain-containing
5_124	1034806	90516	Uncharacterized protein
60_17	1048486	141198	Uncharacterized protein
65_19	1049710	21790	Uncharacterized protein
67_31	1152036	27801	Uncharacterized protein (Fragment)
75_17	1169431	79212	Glycoside hydrolase family 5
9_48	1065541	75159	GFO_IDH_MocA domain-containing protein
9_74	1069652	122002	Glycoside hydrolase family 3

**Figure 8:**
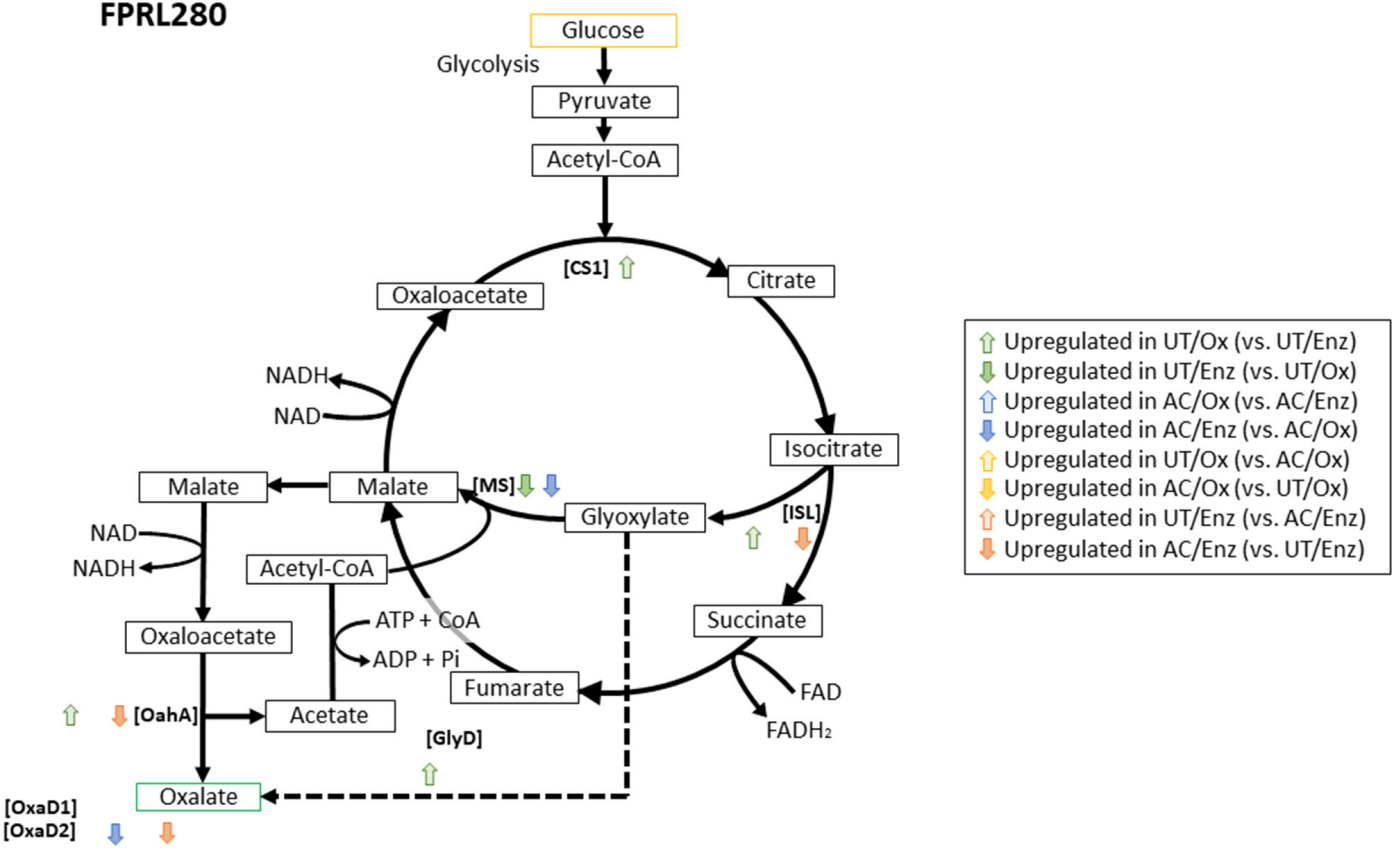
Metabolic mechanisms for the oxalate biosynthesis in the TCA cycle and the GLOX cycle in *R. placenta* (FPRL280). Activities of the involved enzymes: [ISL] Isocitrate lyase; [CS] Citrate synthase; [MS] Malate synthase; [GlyD] Glyoxylate dehydrogenase; [OahA] Oxaloacetase; [OxaD] Oxalate decarboxylase. Modified graphic, based on Munir et al. (2001).

Next, we analyzed the expression of genes that are putatively involved in oxalic acid biosynthesis (for FPRL280 see Figure 8, for MAD-698 and *G. trabeum* see Supplementary Figures 6A,B respectively). Since oxalate plays an important role during early brown rot decay, it is important for the fungi to have working production cycles. We only considered the genes to be upregulated with a log2 fold change ≥ 2 to ensure a significant difference between the comparisons. FPRL280 showed an upregulation of five genes in untreated samples (Figure 8) comparing the two degradation phases. These genes encode isocitrate lyase (ISL; FPRL280_213_3), acetyl CoA-synthase, citrate synthase (CS; FPRL280_520_1), glyoxylate dehydrogenase (GlyD; FPRL280_17_38), as well as an OahA (FPRL280_17_43). MAD-698 upregulated five genes, four of these being orthologs to FPRL280, while *G. trabeum* only upregulated two genes during the oxidative phase, one encoding for an acetyl CoA-synthase and an OahA, also upregulated by FPRL280. Corresponding orthologs were not found for *G. trabeum*, however other genes with these functions can exist in *G. trabeum*.

During enzymatic decay, FPRL280 upregulated a malate synthase-encoding gene (malate synthase; FPRL280_197_5), also expressed by *G. trabeum*, and a malic enzyme, indeed upregulated in MAD-698. MAD-698 additionally upregulated a CS (POSPLADRAFT_ 1058962) and *G. trabeum* an OxaD during enzymatic decay.

Comparing both degradation phases (“Ox” and “Enz”) in acetylated samples revealed an upregulation of a cytochrome c oxidase (FPRL280_30_30) in oxidative samples of FPRL280 and a downregulation of a gene encoding a malic enzyme during enzymatic decay (FPRL280_51_6), also in FPRL280. The other strains did not show significant differences between the samples. When comparing the enzymatic stages of untreated and acetylated samples, an upregulation of genes coding for an OahA (FPRL280_17_43), an ISL (FPRL280_213_3) as well as an acetyl CoA-synthase (FPRL280_74_8) was observed in acetylated samples of FPRL280. MAD-698 did not show significant differences between the treatments for the applied fold change. *G. trabeum* upregulated a malate synthase-encoding gene (GLOTRDRAFT_137388) in untreated samples, as well as an acetyl CoA-synthase like gene (GLOTRDRAFT_ 115850) and an OahA-encoding gene (GLOTRDRAFT_ 115850) in acetylated samples.

All the genes discussed above, the genes which were differentially expressed in all three strains and the genes used in the publication of (Zhang et al., 2016), helped to create a list of 139 genes with putatively important functions during brown rot decay (Supplementary Material 6).

## Discussion

In the present study, we compared the transcriptomes of two strains of *R. placenta* (FPRL280 and MAD-698) as well as another brown rot fungus, *G. trabeum*, during initial and proceeded wood decay, when growing on untreated and acetylated Scots pine sapwood. Differential expression analyses were performed to reach a better understanding of both ongoing processes during brown rot decay, as well as of the inhibitory processes induced by the wood modification.

### Decay Strategies Differ Among All Tested Strains

The presented data strongly suggest that the phenotypic differences that have been reported between the two *R. placenta* strains (MAD-698 and FPRL280) (Thaler et al., 2012; Kölle et al., 2020) can be explained to a large extent by regulatory differences. In total, MAD-698 expressed around 1,000 more genes than FPRL280 across all treatments (Supplementary Figure 2) during wood degradation. Regarding CAZyme-encoding genes, the overall expressed gene set appeared quite similar during oxidative and enzymatic decay. However, a small number of genes showed dramatically differing expression levels, such as the endoglucanase Cel12A-encoding gene (FPRL280_170_10; POSPLADRAFT_1071715; Figure 3), which is an important protein during enzymatic brown rot decay (Ryu et al., 2011; Zhang et al., 2016; Umezawa et al., 2020) and was also highly differentially regulated between oxidative and enzymatic phases in all three tested fungi.

All three strains displayed a switch from the oxidative to the enzymatic decay stage, which was characterized by a clear shift in the gene expression profiles (Figure 2) supporting the theory of a two-stepped brown rot decay (Eaton and Hale, 1993; Goodell, 2003; Fackler et al., 2010; Arantes et al., 2012; Alfredsen et al., 2016; Ringman et al., 2016; Zhang et al., 2016; Beck et al., 2018). The functional annotation of the differentially expressed genes also differed between the two degradation stages. While more cytochrome P450s were upregulated during oxidative decay, aldo-keto reductases, transporters, proteases and genes encoding for proteins involved in sugar metabolism were upregulated during the enzymatic stages (Figure 2; Supplementary Material 4). In particular five CAZyme-encoding genes displayed a very strong induction in the enzymatic phase: the genes coding for the endoglucanases Cel5A, Cel5B, and Cel12A (for all three strains) as well as for the two CE16 carbohydrate esterases (for *R. placenta*) (FPRL280_428_3, FPRL280_27_40; POSPLADRAFT_1067618, POSPLADRAFT_1179218) (Figure 3), supporting previous findings (Martinez et al., 2009; Vanden Wymelenberg et al., 2010; Ryu et al., 2011; Beck et al., 2018). Six other genes were significantly upregulated during oxidative decay in all three strains, including three uncharacterized genes, one encoding for an aspartate aminotransferase, one serine/threonine-protein kinase and one cyclin N-terminal domain-containing protein. These genes are clear candidates as common biomarkers for detection and monitoring of brown rot decay in untreated wood.

FPRL280 was shown to have a slower initial growth speed than MAD-698, while producing a higher mass loss during the 1st days of degradation (Thaler et al., 2012; Kölle et al., 2020). This might indicate that FPRL280 is able to recognize the substrate and start the degradation earlier than MAD-698 (Kölle et al., 2020). In line with these observations, FPRL280 was found to upregulate more CAZy and BRS genes during the initial, oxidative decay phase than MAD-698 (Figure 5). MAD-698, on the other hand, was found to express an overall higher number of CAZy encoding and BRS genes, albeit at lower levels, compared to FPRL280 (Figures 2, 3), supporting the enzymatic degradation with more BRS “Ox” genes (Figure 4). These results, including the higher degradation rates found by Kölle et al. (2020), lead to the conclusion that the degradation approach in MAD-698 is the more effective one.

Looking at highly differentially expressed genes (log2 FC ≥ 6) between both strains of *R. placenta* during the initial, oxidative decay, FPRL280 upregulated more proteins with protease function, while the largest functional group in MAD-698 was cytochrome P450s (Supplementary Material 5). Cytochrome P450s are likely involved in reactions required for the biosynthesis of methoxyhydroquinones, as well as for hydroxylation of lignin fragments, which is important for lignocellulolytic processes (Ide et al., 2012) and could support the more effective degradation observed in MAD-698. An additional interesting gene found to be highly upregulated in MAD-698 encodes a citrate synthase (FPRL280_520_1/ POSPLADRAFT_1156738), which is assumed to play an important role in the oxalate cycle (Munir et al., 2001) and could suggest that oxalate production is more active in MAD-698 than in FPRL280. Together, these findings further strengthen the assumption that the two strains follow different regulatory routes.

*G. trabeum* seems to proceed with a radically different decay strategy than *R. placenta*. Overall, *G. trabeum* appears to be a more aggressive degrader growing on Spruce [*Picea abies* (L.) Karst.], since it was demonstrated that it needs only 28 days to gain a 16% mass loss, while *R. placenta* reached the same mass loss only after 56 days (Fackler et al., 2010). Surprisingly, however, although *G. trabeum* has a wider range of CAZyme-encoding genes in its genome (Floudas et al., 2012; Gaskell et al., 2017), these were not induced to the same extent from early to late decay stage as in *R. placenta* (Figure 3). Previous research showed that *G. trabeum* might have different carbohydrate preferences than *R. placenta* (Presley et al., 2018). In addition to this, it was also shown that *G. trabeum* expressed more oxidoreductases during late decay compared to early decay stages (Presley et al., 2018). This observation could not be confirmed in our data, where *G. trabeum* expressed a similar number of genes with oxidoreductase activity during both phases, while *R. placenta* expressed more oxidoreductases during enzymatic decay (especially MAD-698). It has to be noted, however, that the test setup used in our study was developed for *R. placenta* and might not optimally capture the same degradation stages for *G. trabeum*, and thus it is possible that the enzymatic phase was not yet completely induced in our late time point samples.

### Wood Acetylation Leads to Genome-Wide Expression Changes

Wood acetylation led to differential expression of several hundred genes in each degradation stage and in all three fungi (Figures 1B,C) indicating that the fungi are clearly reacting to the modification on a genome-wide scale. Previously, a group-wise upregulation of four oxidative genes [glucose oxidase (GOx2, CAZy family AA5), copper radical oxidase (Cro1, CAZy family AA3), alcohol oxidase 2 and 3 (AlOx2, AlOx3; CAZy family AA3)] was shown (Kölle et al., 2019). All corresponding proteins are putatively involved in the extracellular H_2_O_2_ production. This group-wise upregulation was observed on wood samples with increasing levels of acetylation (to a maximum level of 20%), compared to untreated samples during oxidative decay (Kölle et al., 2019). This was confirmed in the present study for GOx2 by showing a clear upregulation of the corresponding ortholog in the acetylated samples in all three tested fungi (FPRL280_46_12; POSPLADRAFT_1040696; GLOTRDRAFT_139980). Upregulation of AlOx3 in the acetylated samples was also observed in transcriptomic data for FPRL280 and MAD-698 (FPRL280_40_14; POSPLADRAFT_1183136). Since these genes behave similarly for all three brown rot fungi and their importance during early brown rot decay have been proven, they could also be suitable as potential biomarkers for brown rot degradation in acetylated wood. Notably, proteins putatively involved in the oxalic acid synthesis were not as differentially expressed in acetylated samples between the two phases as in untreated samples, suggesting an impaired oxalate cycle due to the acetylation. This information could be an important part of the puzzle understanding the mode of action of acetylated wood. Furthermore noteworthy is the upregulation of an iron permease-encoding gene (FPRL280_195_6) in acetylated samples, since it seems to be upregulated during the enzymatic phase in untreated samples and is also strongly upregulated in acetylated samples in the oxidative decay phase.

Surprisingly, more genes related to sugar metabolism were upregulated in acetylated samples during oxidative decay in all three strains (Supplementary Material 4). An upregulation of these genes is typically part of the subsequent enzymatic decay phase and induced by polysaccharides solubilized during initial decay (Zhang and Schilling, 2017). Nevertheless, the accumulated expression levels of these genes are rather low in acetylated samples—even in comparison to untreated samples during oxidative decay, and therefore resemble more of a scouting reaction. Previous studies demonstrated an extremely slow mass loss on acetylated samples during early decay (Alfredsen et al., 2016; Ringman et al., 2017; Beck et al., 2018), which was attributed to the protective function of the acetylation. However, this starving situation apparently induces a scouting reaction leading to the production of a broader range of enzymes involved in sugar metabolism (Van Munster et al., 2014). Nevertheless, the affected gene complements of the different strains varied in composition, showing that different groups of genes were either enhanced or inhibited by the acetylation. This further underlines the fact that the strains not only follow different decay strategies, but also seem to react differently to the modification. Expression profiles of all three strains in acetylated samples were furthermore dominated by many uncharacterized genes. Finding the functions of these genes might be crucial for the understanding on how acetylation inhibits fungal growth.

All three tested fungi displayed a high number of genes that were heavily affected by the acetylation treatment. Hierarchical clustering revealed that in total, more than 50% of the genes in all three strains are located in clusters that are more or less affected by the acetylation, showing the broad genome-wide effects of the modification. A very interesting observation was a cluster with genes in *G. trabeum* that were enhanced in AC/Enz samples, containing many BRS “Ox” genes as well as closely located TFs. This strongly indicates that *G. trabeum* did not reach a proper enzymatic degradation phase in acetylated samples. This phenomenon was not seen in *R. placenta*, demonstrating the differences in decay strategies and/or timing of the responses and the use of different approaches to overcome the inhibition associated with the wood modification.

In addition to this, *G. trabeum* upregulated eight hydrophobin-encoding genes in the oxidative degradation phase on acetylated wood. This might represent a stress-induced response, since hydrophobins are for example involved in the formation of fruiting bodies and aerial hyphae (Wessels et al., 1991). Filamentous fungi might also use hydrophobins for better adherence to hydrophobic surfaces (Wessels, 1996). In a previous study on the wettability and swelling of treated wood (Moghaddam et al., 2016), it was shown that acetylation renders the wood surface (of fresh cut veneers) more hydrophobic, which might explain the observed response.

Considering CAZyme-encoding genes, the majority of these were found in gene clusters affected by the acetylation in all three strains. Notably, a gene cluster including many BRS genes could be identified that was strongly affected in FPRL280 (Cluster 20) and *G. trabeum* (Cluster 7), albeit less so in MAD-698 (Cluster 14). Besides these, particularly AA3_2-and GT-encoding genes were identified in clusters that demonstrated a strong inhibition by acetylation (e.g., Cluster 1 in FPRL280). As already discussed, proteins from the AA3_2 family are important during brown rot decay and known to support cellulases and hemicellulases. GTs are, among others, necessary for the biosynthesis of the fungal cell wall (Klutts et al., 2006), indicating that the formation of new mycelium is either inhibited by the acetylation or the lack of energy leads to decreased GT production.

The clusters containing genes that were affected by acetylation also contained higher percentages of TFs. Many TFs clustered closely to important CAZy-encoding genes or genes related to oxidative brown rot decay, indicating that these might be interesting targets for further studies aimed at gaining a better molecular understanding of brown rot gene regulation.

### Genes Relevant for Brown Rot Decay

Previous studies have presented a wide range of genes that appear to be involved in brown rot decay (Martinez et al., 2009; Vanden Wymelenberg et al., 2010; Ryu et al., 2011; Zhang et al., 2016; Beck et al., 2018). We created a list of relevant BRS genes including previously identified genes and genes newly found in this study (Supplementary Material 6). Especially genes that were similarly expressed in all three strains during either the oxidative or the enzymatic degradation phase (Table 1) could be of interest for future studies or to be used as BRS biomarkers. Similar to the comparison of expression levels, this gene list contains a high number of uncharacterized genes (oxidative phase 3/6, enzymatic phase 8/19), highlighting the importance of further annotations.

The classification into BRS “Ox” and BRS “Enz” genes was conducted based on their expression data and their respective functions. The hierarchical clustering revealed many clusters related to enzymatic degradation, in which BRS “Ox” genes were located together with BRS “Enz” genes, indicating co-regulation despite differing functions. For future research, a phase-specific classification into “early” and “late response” might be more suitable than a function-based classification.

## Conclusions

Gaining more insight into the two-stepped decay mechanism of brown rot fungi was one of the reasons to compare the transcriptomes of the two different strains of *R. placenta* (FPRL280 and MAD-698) and *G. trabeum*. The results confirm the presence of a two-stepped degradation mechanism in all three fungi. However, the underlying regulatory switch seems dynamic rather than a rigid bipartite system and different strains use varying compositions of genes active in parallel during oxidative and enzymatic decay. These findings indicate that the duration of the early phase, dominated by oxidative processes, and the start of the later phase, dominated by enzymatic processes, are highly strain-specific, as well as the transition time between both phases. Nevertheless, genes expressed during initial and later decay by all three strains could be used as biomarkers for the respective degradation phase.

Wood modification by acetylation had a profound effect on gene expression patterns on a genome-wide level. Overall, more genes were found to be upregulated on acetylated wood, such as CAZymes, but appeared to be attenuated in accordance with a starvation stress-induced scouting-like response. Glucose oxidase (GOx2) was upregulated in acetylated samples in all three strains, making it a promising biomarker for the investigation of acetylation effects. The vast number of differentially expressed uncharacterized genes identified in this study highlights the importance of future efforts to enrich the annotation of brown rot fungal genomes.

## Data Availability Statement

The datasets presented in this study can be found in online repositories. The names of the repository/repositories and accession number(s) can be found below: https://www.ncbi.nlm.nih.gov/, PRJNA681134.

## Author Contributions

The research was initiated and designed by AP, MK, JB, and MC. MK and MC performed the analyses. MK and AP co-wrote the paper with support of MC and JB. All authors were included in the interpretation of the data, read and approved the final manuscript.

## Conflict of Interest

The authors declare that the research was conducted in the absence of any commercial or financial relationships that could be construed as a potential conflict of interest.

## Publisher's Note

All claims expressed in this article are solely those of the authors and do not necessarily represent those of their affiliated organizations, or those of the publisher, the editors and the reviewers. Any product that may be evaluated in this article, or claim that may be made by its manufacturer, is not guaranteed or endorsed by the publisher.
