# Supplementary material for: Comparative Transcriptomics During Brown Rot Decay in Three Fungi Reveals Strain-Specific Degradative Strategies and Responses to Wood Acetylation
Source: Front Fungal Biol. 2021 Sep 6;2:701579. doi: 10.3389/ffunb.2021.701579 (PMC10512373; doi:10.3389/ffunb.2021.701579)
Supplement: Supplementary file 1 [file Data_Sheet_1.PDF]

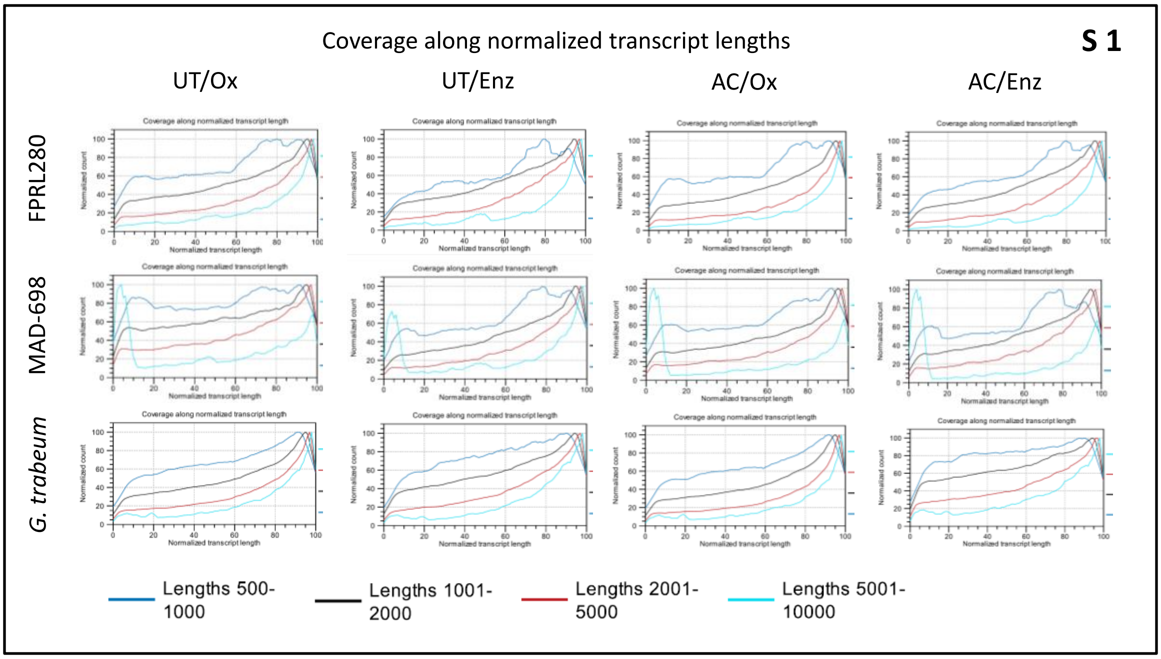

Supplemental Figure 1 Distribution of fragment lengths for *Rhodonia placenta* FPRL280 and MAD-698 and *Gloeophyllum trabeum* for all treatments (UT and AC) and decay stages (Ox and Enz).

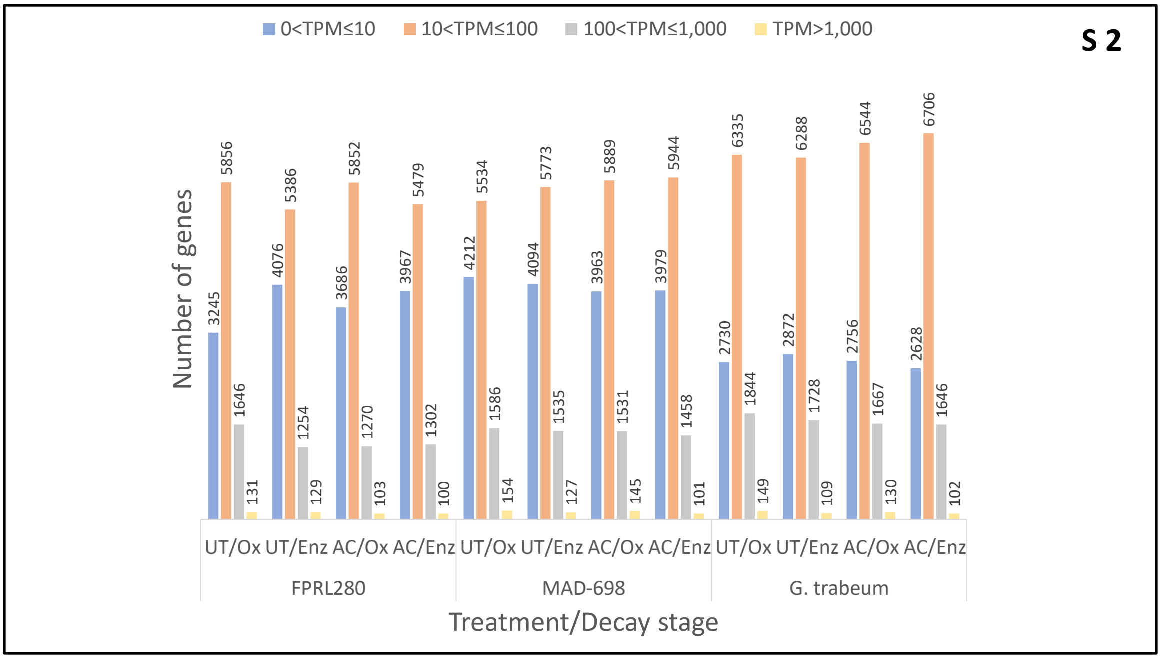

Supplemental Figure 2 Number of expressed genes in each treatment in the three strains used in this study: *Rhodonia placenta* (FPRL280 and MAD-698) and *Gleophyllum trabeum*, using different cutoffs (0<TPM≤10, 10<TPM≤100, 100<TPM≤1,000, TPM>1,000). Included are the two treatments untreated (UT) and acetylated (AC), as well as two degradation stages oxidative (Ox) and enzymatic (Enz).

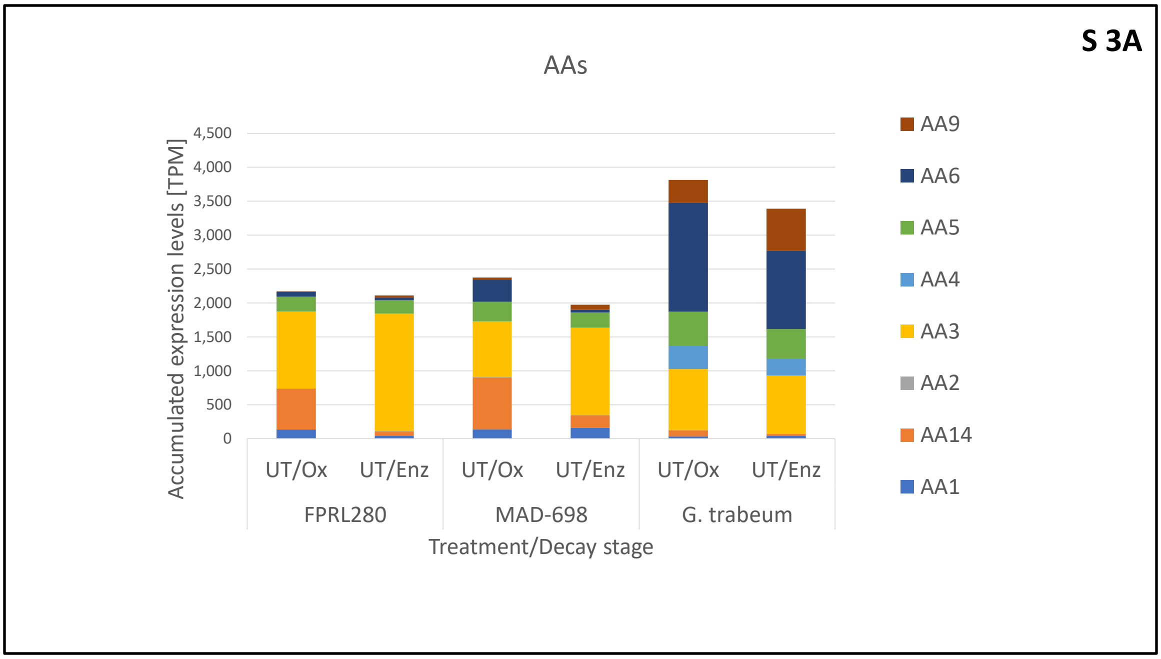

Supplemental Figure 3A Accumulated expression values (transcripts per million (TPM)) for the CAZy family AA in strains of *Rhodonia placenta* and *Gloeophyllum trabeum* showing the differences between the strains and the decay stages.

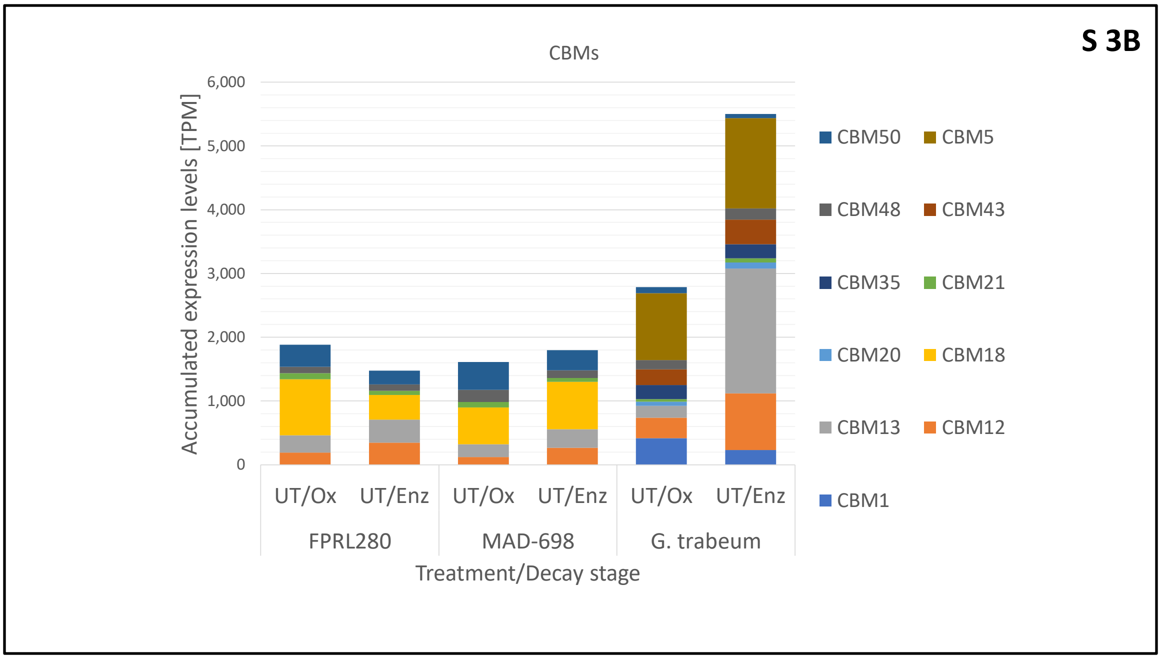

Supplemental Figure 3B Accumulated expression values (transcripts per million (TPM)) for the carbohydrate binding modules (CBMs) in strains of *Rhodonia placenta* and *Gloeophyllum trabeum* showing the differences between the strains and the decay stages.

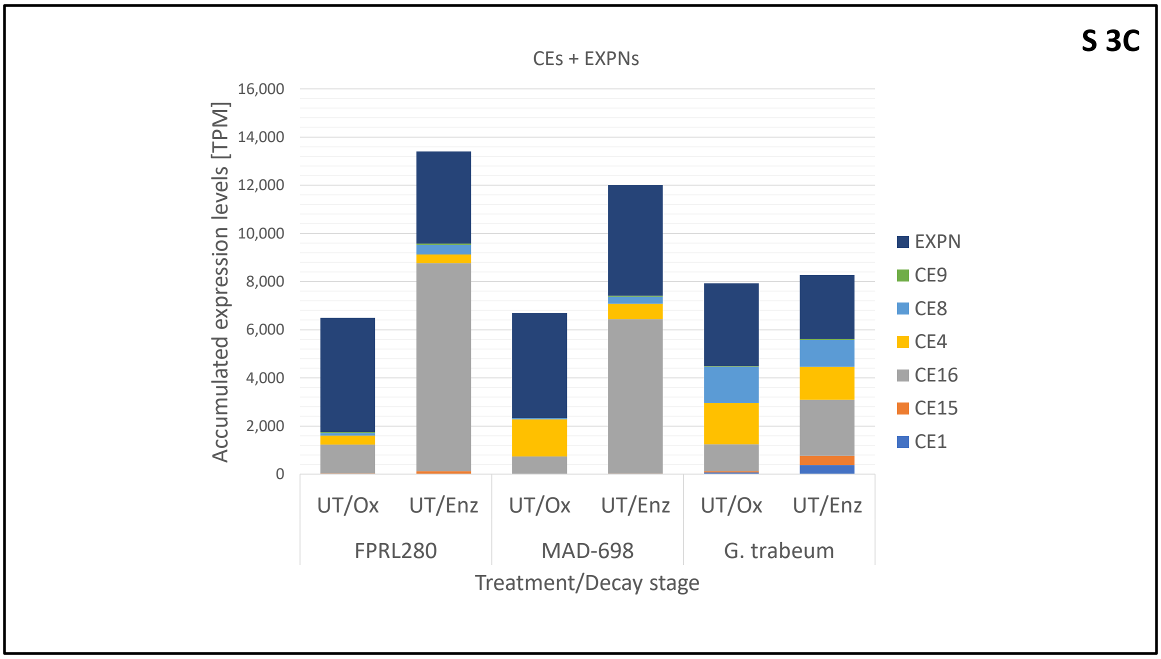

Supplemental Figure 3C Accumulated expression values (transcripts per million (TPM)) for the CAZy family CE and expansins in strains of *Rhodonia placenta* and *Gloeophyllum trabeum* showing the differences between the strains and the decay stages.

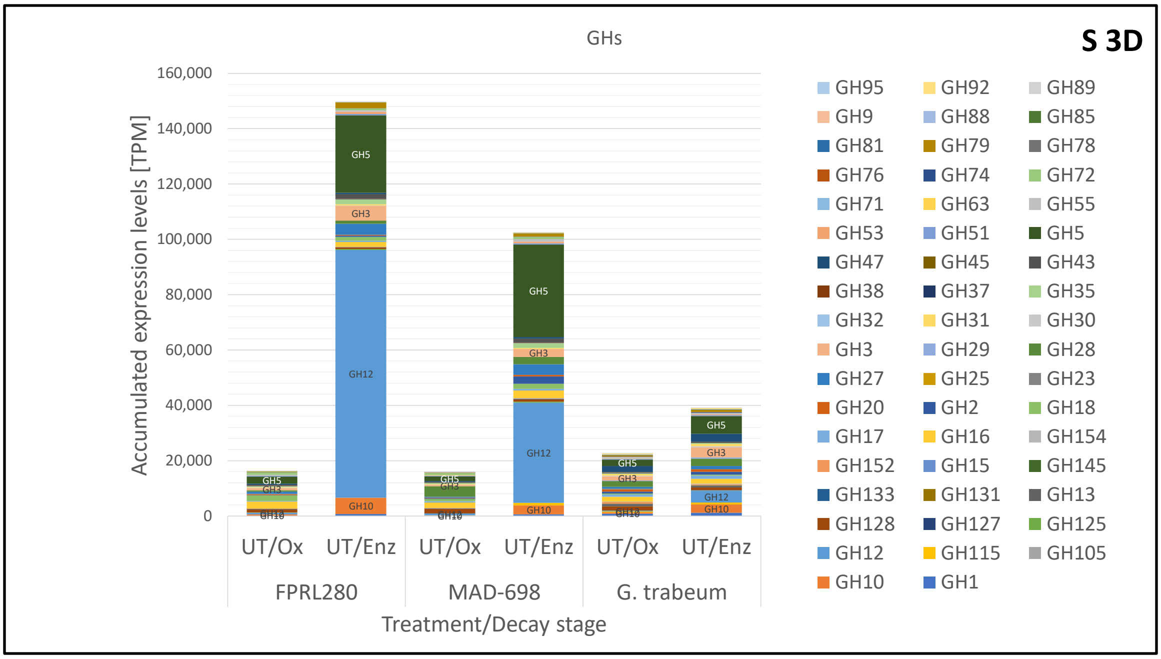

Supplemental Figure 3D Accumulated expression values (transcripts per million (TPM)) for the CAZy family GH in strains of *Rhodonia placenta* and *Gloeophyllum trabeum* showing the differences between the strains and the decay stages.

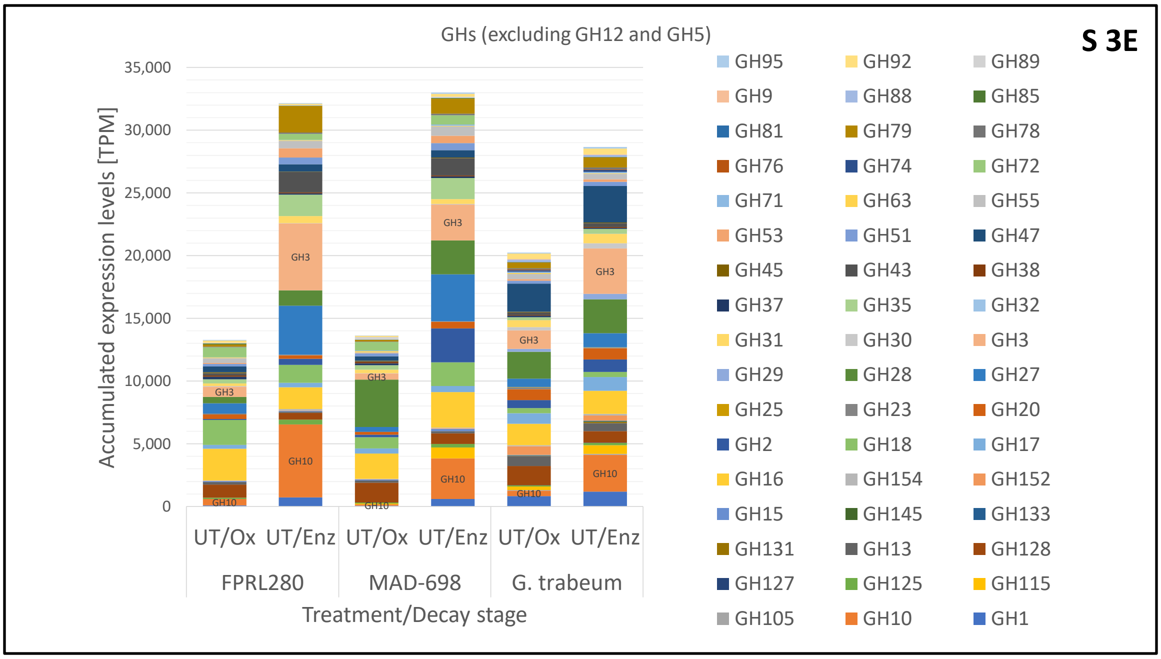

Supplemental Figure 3E Accumulated expression values (transcripts per million (TPM)) for the CAZy family GH in strains of *Rhodonia placenta* and *Gloeophyllum trabeum* showing the differences between the strains and the decay stages. The families of GH12 and GH5 are excluded, for enhanced visibility of the remaining groups.

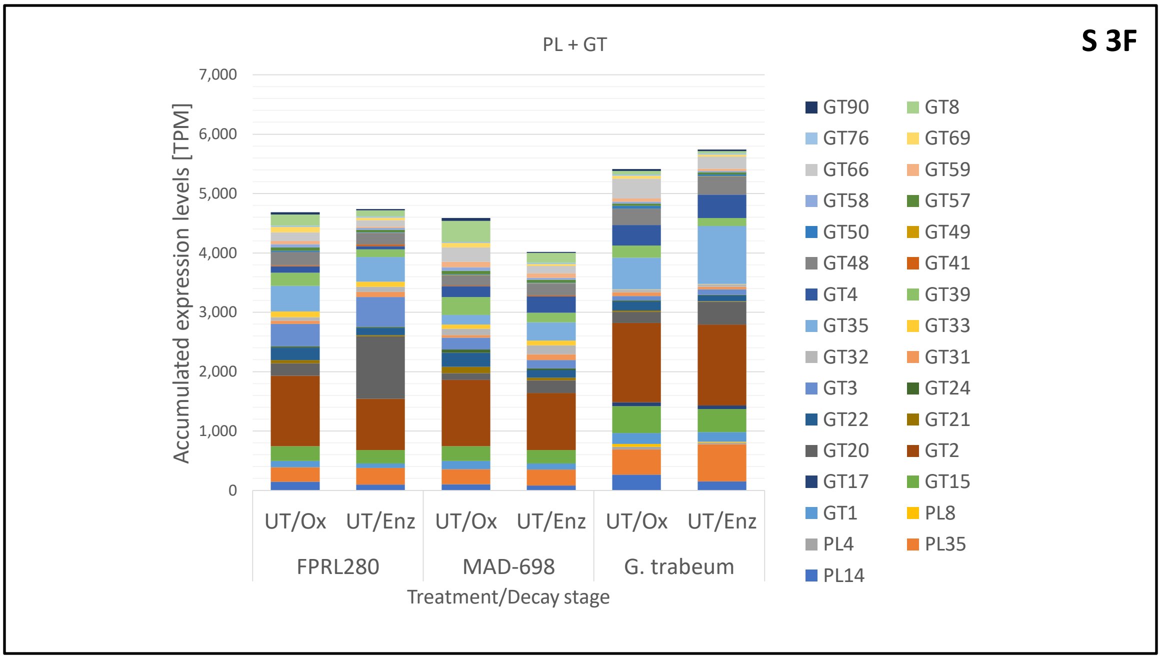

Supplemental Figure 3F Accumulated expression values (transcripts per million (TPM)) for the CAZy families PL and GT in strains of *Rhodonia placenta* and *Gloeophyllum trabeum* showing the differences between the strains and the decay stages.

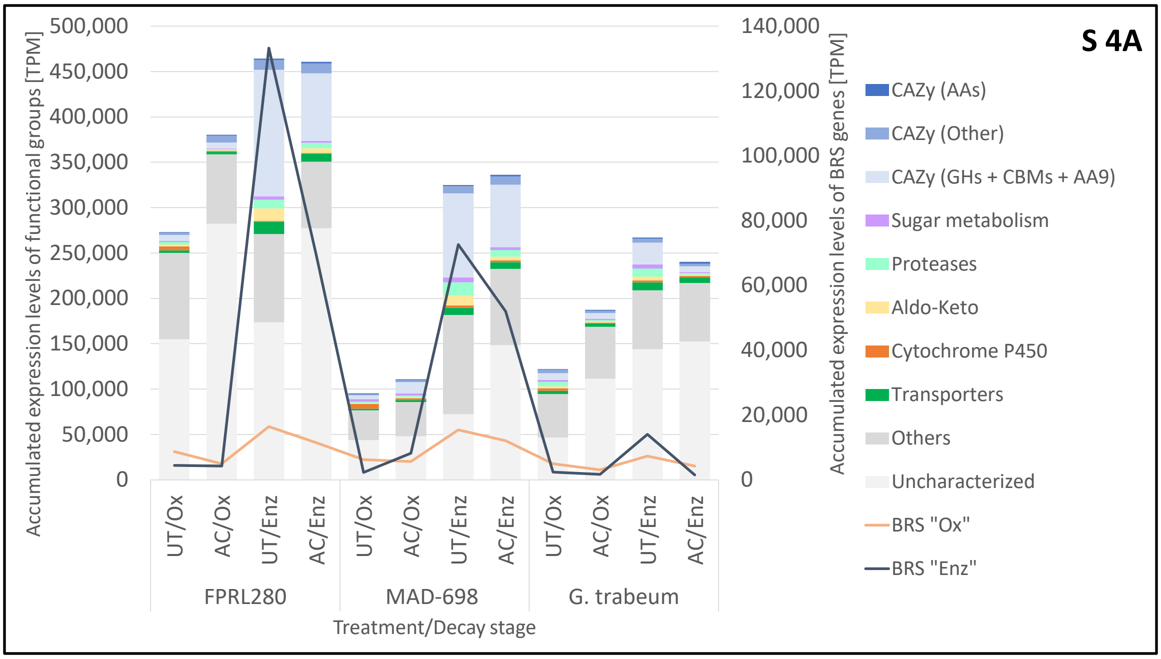

Supplemental Figure 4A TPMs of the different groups are shown with bars in different colors. The graphic shows the results for the differences between untreated and acetylated samples during the oxidative and the enzymatic phase for the two strains of *R. placenta.* and *G. trabeum.*

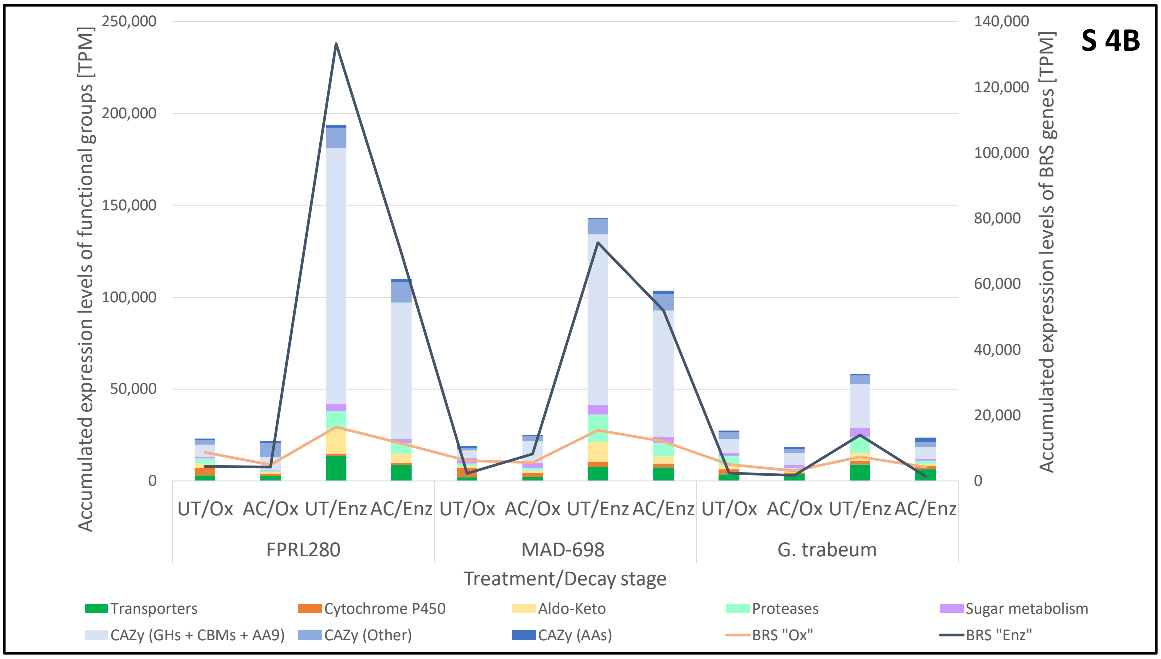

Supplemental Figure 4B TPMs of the different groups are shown with bars in different colors. The graphic shows the results for the differences between untreated and acetylated samples during the oxidative and the enzymatic phase for the two strains of *R. placenta.* and *G. trabeum*. Groups with “Uncharacterized” genes, as well as genes belonging to the category of “Others” are excluded.

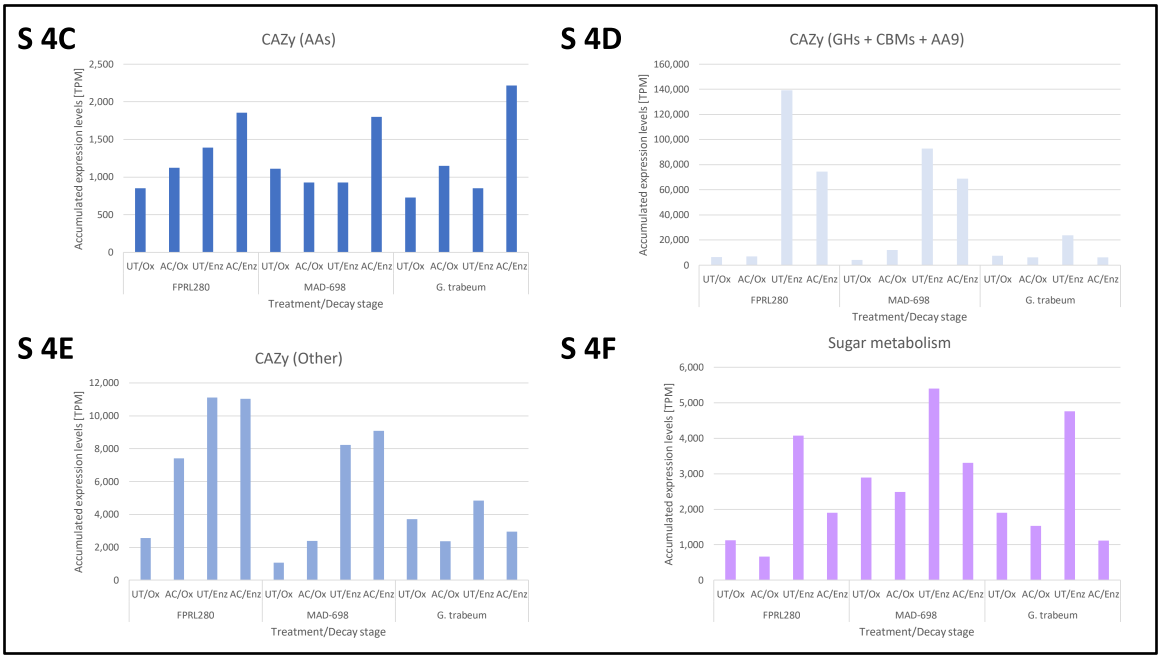

Supplemental Figure 4C-F TPMs of the different groups are shown separately: **C**: CAZy family AA; **D**: CAZy families GH and AA9 + carbohydrate binding modules (CBMs); **E**: other CAZy families; **F**: sugar metabolism. The graphic shows the results for the differences between untreated and acetylated samples during the oxidative and the enzymatic phase for the two strains of *R. placenta.* and *G. trabeum.*

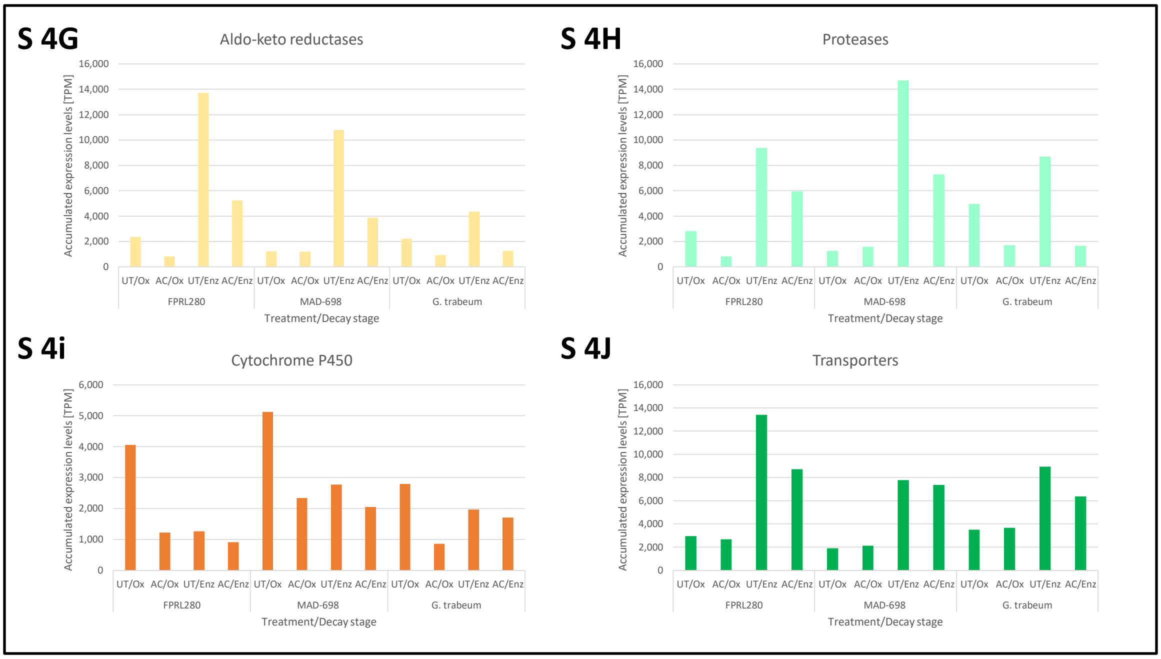

Supplemental Figure 4G-J TPMs of the different groups are shown separately: **G**: Alfo-keto reductases; **H**: Proteases; **i**: Cytochrome P450; **J**: Transporters. The graphic shows the results for the differences between untreated and acetylated samples during the oxidative and the enzymatic phase for the two strains of *R. placenta.* and *G. trabeum.*

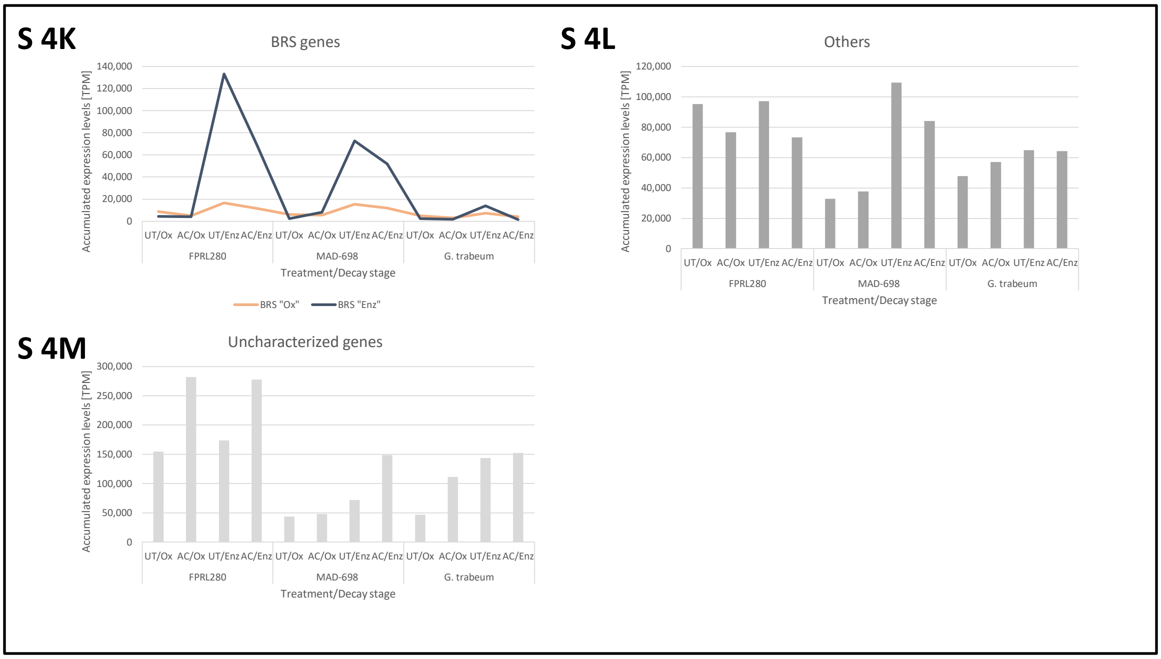

Supplemental Figure 4K-M TPMs of the different groups are shown separately: **K**: BRS genes; **L**: Others; **M**: Uncharacterized genes. The graphic shows the results for the differences between untreated and acetylated samples during the oxidative and the enzymatic phase for the two strains of *R. placenta.* and *G. trabeum.*

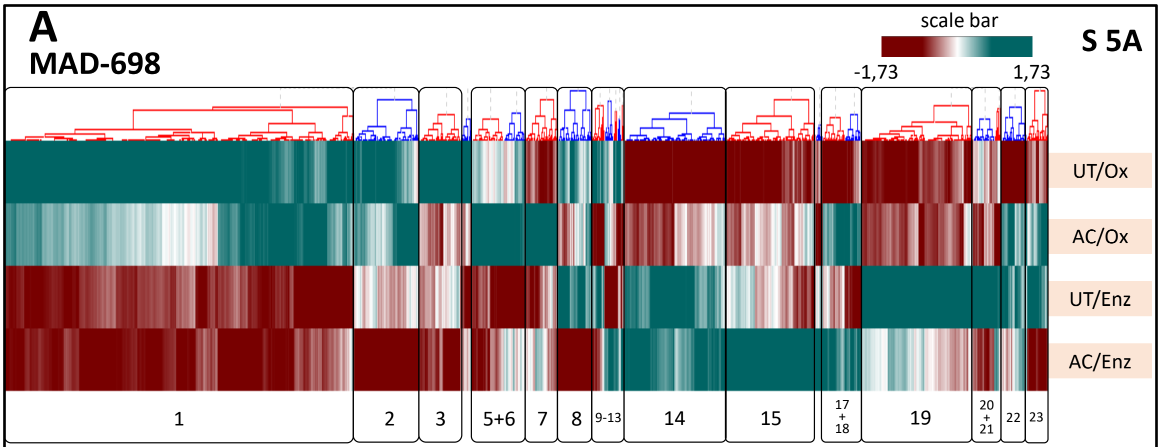

B

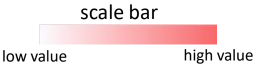

Clusters	1	2	3	4	5	6	7	8	9	10	11	12	13	14	15	16	17	18	19	20	21	22	23
TFs	26.97	2.25	3.37	0	1.12	7.87	6.74	3.37	1.12	1.12	1.12	1.12	0	14.61	8.99	0	3.37	1.12	10.11	1.12	1.12	1.12	2.25
BRS "Ox"	18.28	1.08	10.75	1.08	0	2.15	2.15	0	1.08	1.08	0	0	0	10.75	13.98	1.08	1.08	4.30	26.88	1.08	1.08	2.15	0
BRS "Enz"	0	5	0	0	0	0	0	0	0	0	0	0	0	40	5	0	0	0	45	0	0	5	0
CAZy	26.04	1.51	2.64	0.75	2.26	1.89	2.64	2.26	0.38	0.38	0.38	0.38	0.38	15.85	19.25	0.38	2.64	0.75	13.58	1.51	0.75	2.64	0.75

Supplemental Figure 5A Results for the hierarchical clustering of the transcriptomes of *R. placenta* MAD-698 in all tested conditions. **A** UT/Enz: untreated samples during enzymatic decay; AC/Enz: acetylated samples during enzymatic decay; UT/Ox: untreated samples during oxidative decay; AC/Ox: acetylated samples during oxidative decay. **B** Heat map of brown rot specific genes (BRS), CAZy genes and transcription factors (TFs). The numbers represent the percentage [%] of the abundance of genes of the respective group. To annotate TFs, different references (Zhang et al. 2019, Martinez et al. 2009, UniProt, MycoCosm) were used and the respective orthologs from *R. placenta* and *G. trabeum* identified.

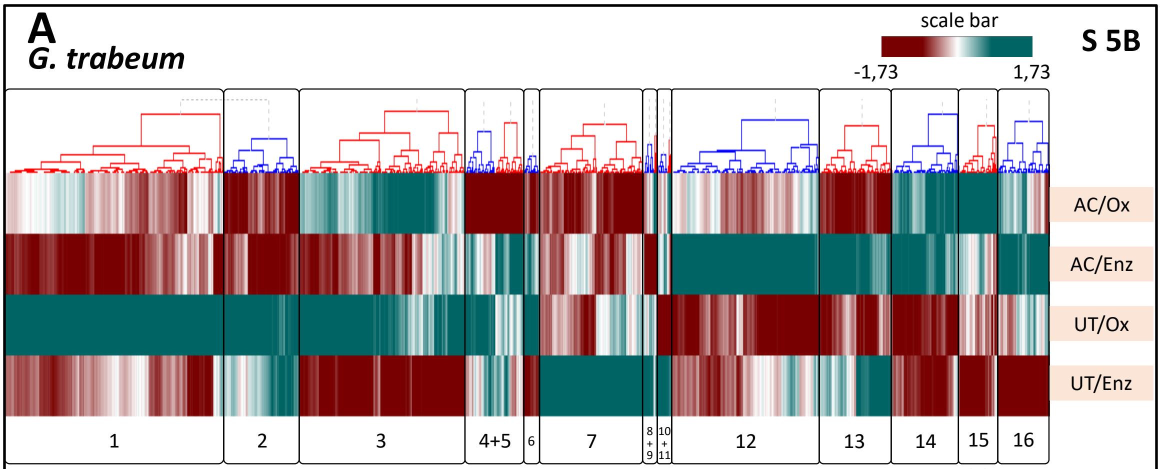

B

scale bar
low value high value

Cluster	1	2	3	4	5	6	7	8	9	10	0	12	13	14	15	16
TFs	14.56	5.83	5.83	3.88	4.85	2.91	12.62	2.91	0	0	0	21.36	8.74	5.83	0	10.68
BRS "Ox"	13.11	11.48	11.48	0	0	0	26.23	9	0	1.64	0	21.31	1.64	4.92	1.64	6.56
BRS "Enz"	5	10	0	0	0	0	70	5	5	0	0	5	0	0	0	0
CAZy	12.7	7.30	13.02	1.9	1.27	1.59	22.86	4.44	1.9	0.95	0.32	11.43	5.4	6.98	4.44	3.49

Supplemental Figure 5B Results for the hierarchical clustering of the transcriptomes of *G. trabeum* in all tested conditions. **A** UT/Enz: untreated samples during enzymatic decay; AC/Enz: acetylated samples during enzymatic decay; UT/Ox: untreated samples during oxidative decay; AC/Ox: acetylated samples during oxidative decay. **B** Heat map of brown rot specific genes (BRS), CAZy genes and transcription factors (TFs). The numbers represent the percentage [%] of the abundance of genes of the respective group. To annotate TFs, different references (Zhang et al. 2019, Martinez et al. 2009, UniProt, MycoCosm) were used and the respective orthologs from *R. placenta* and *G. trabeum* identified.

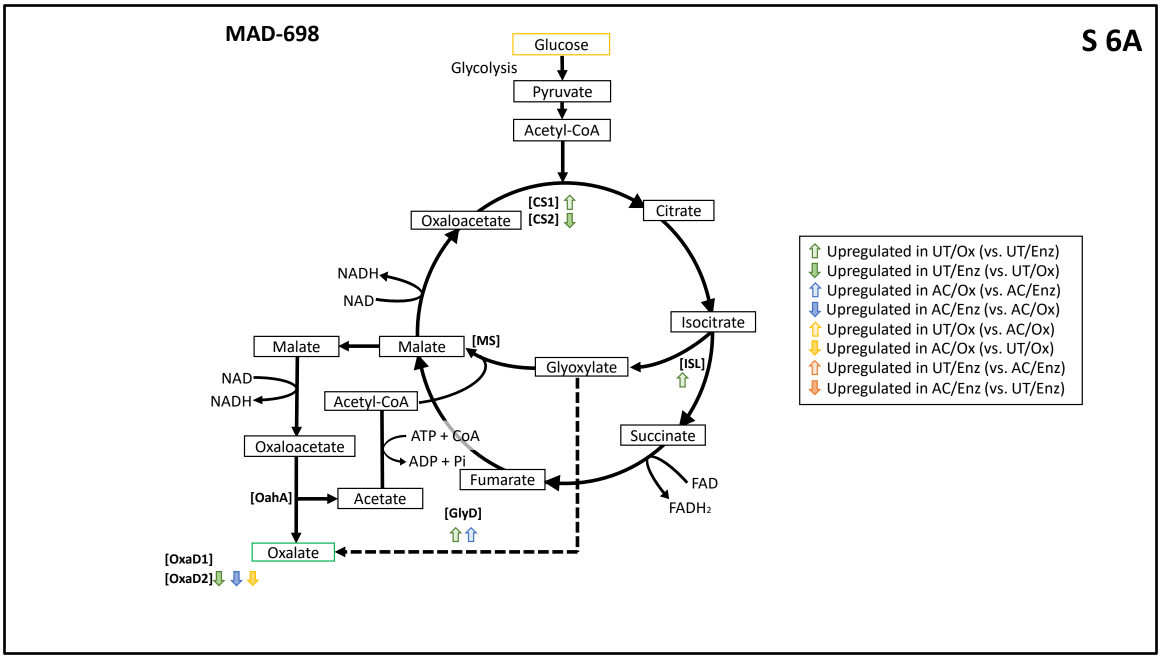

Supplemental Figure 6A Metabolic mechanisms for the oxalate biosynthesis in the TCA and the GLOX cycle in *Rhodonia placenta* (MAD-698). Activities of the involved enzymes: [ISL] Isocitrate lyase; [CS] Citrate synthase; [MS] Malate synthase; [GlyD] Glyoxylate dehydrogenase; [OahA] Oxaloacetase (OahA); [OxaD] Oxalate decarboxylase. Modified graphic, based on Munir *et al.* (2001).

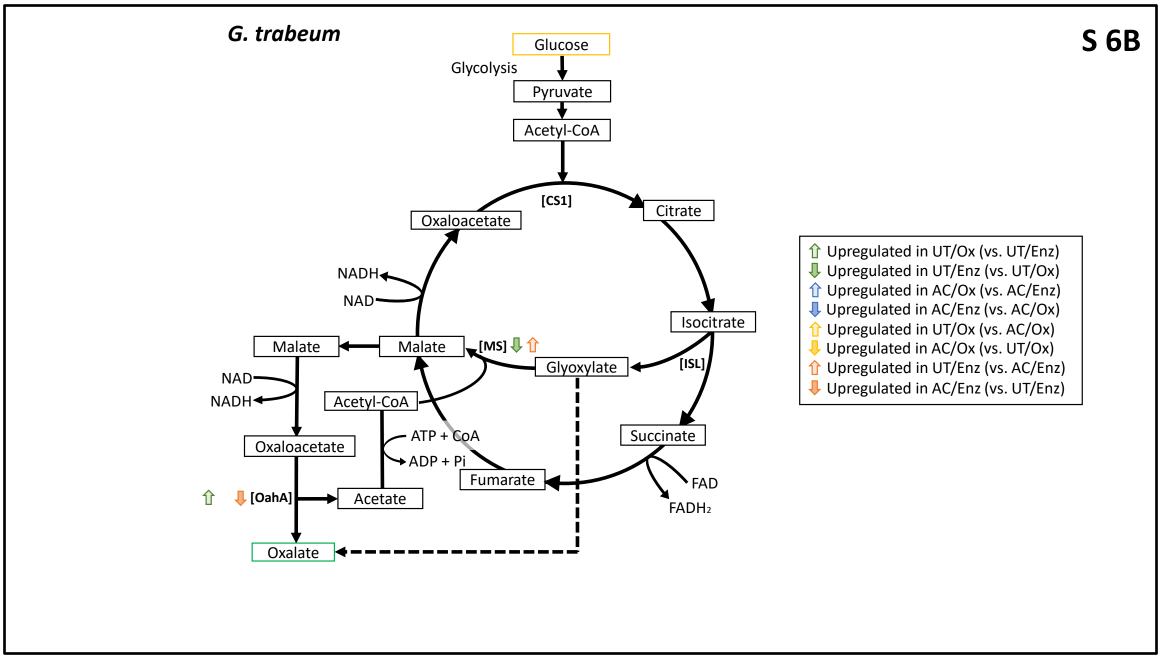

Supplemental Figure 6B Metabolic mechanisms for the oxalate biosynthesis in the TCA and the GLOX cycle in *G. trabeum* (MAD-698). Activities of the involved enzymes: [ISL] Isocitrate lyase; [CS] Citrate synthase; [MS] Malate synthase; [GlyD] Glyoxylate dehydrogenase; [OahA] Oxaloacetase (OahA); [OxaD] Oxalate decarboxylase. Modified graphic, based on Munir *et al.* (2001).
